# Late adolescents entering college intending a career as police officers hold more right-leaning views than their peers

**DOI:** 10.1073/pnas.2500220122

**Published:** 2025-08-06

**Authors:** Tyler T. Reny, Marcel F. Roman, Benjamin J. Newman, David O. Sears

**Affiliations:** ^a^Department of Politics and Policy, Claremont Graduate University, Claremont, CA 91711; ^b^Department of Government, Harvard University, Cambridge, MA 02138; ^c^School of Public Policy and Department of Political Science, University of California-Riverside, Riverside, CA 92507; ^d^Department of Political Science and Department of Psychology, University of California-Los Angeles, Los Angeles, CA 90095

**Keywords:** policing, public opinion, political attitudes, criminal justice, political psychology

## Abstract

One longstanding explanation for bias and excessive force in policing is selection—the assertion that those who select to work in law enforcement are more likely to hold far-right, intolerant, and antiegalitarian, views than the public. While intuitive and widely believed, the evidence in support of the selection hypothesis is surprisingly thin. We offer a vital contribution to the literature by exploring selection at one of its earliest phases: late adolescents entering college and the career-formulation stage of life. Utilizing surveys of over 13 million late adolescents collected across 44 y in the United States, we find that those intending a career as a law enforcement officer hold more right-leaning views on race relations, immigration and intercultural relations, women and gender roles, homosexuality and gay rights, drug-use, freedom of speech, and criminal justice, than their peers intending other careers or their modal peer who is undecided with respect to career intentions and serves as a “blank slate” comparison group. Critically, these findings largely hold among Whites and non-Whites but are somewhat attenuated among women. These sources of heterogeneity are noteworthy given evidence suggesting that hiring more non-White officers may ameliorate bias and excessive force in policing and accompanying pushes to diversify police forces. Our findings add caution to such pushes by suggesting that the non-Whites who select into law enforcement may hold views that reproduce, vs. attenuate, existing police bias. In contrast, our findings direct reform efforts toward expanding the ranks of female officers, bias testing, and training during police recruitment.

## Significance

Prior research finds that police officers hold more right-wing social and political views than the general public. Using a survey of over 13 million late-adolescent Americans entering college collected across 44 y, we offer evidence that those intending a career in law enforcement hold more right-leaning social and political views than their peers. This relationship between career intentions and right-leaning views is somewhat reduced when focusing on non-Whites but more notably reduced when focusing on women. Our findings suggest that, in order to reduce bias in policing, it may be necessary to alter or enlarge the pool of candidates typically entering the academy and to implement screening and training of candidates in early stages of the police recruitment process.

In addition to documented bias against ethno-racial minorities (1, 2), the scholarly literature offers evidence of unfavorable police treatment of immigrants (3, 4), women (5, 6), gay and transgender individuals (7, 8), and the poor (9, 10). In short, in societies with multifaceted stratification, decades of scholarship uncover bias in policing against various lower status and minority groups. While scholarship offers several explanations for the sources of this bias, one longstanding hypothesis points to the type of people who select into police work in the first place. This selection hypothesis posits that people who choose a career in law enforcement harbor far-right, intolerant, and antiegalitarian social and political views (11–13).

The selection hypothesis is grounded in literature on personality, social, and organizational psychology yielding person-organization congruence theory (14). This theory contends that people seek out and thrive in organizations that suit their inclinations, motivations, and worldviews (15). Individuals who uphold group-based social hierarchies are argued to locate themselves and flourish in “hierarchy-enhancing” institutions that produce or reinforce the subordination of lower status groups (16). These institutions are defined as those that maintain social hierarchy and intergroup inequality by making “disproportionately greater positive social allocations (or fewer negative social allocations) to dominant groups than subordinate groups” (14). A prime example of hierarchy-enhancing institutions are law enforcement agencies in the United States, which historically functioned to maintain racial caste systems (17) and continue to operate with systemic bias toward ethno-racial and other minority groups (1, 2). In short, this framework contends that people who gravitate toward employment in law enforcement endorse social hierarchy and harbor animus toward subordinate social groups.

The selection hypothesis frequently underlies academic and popular discourse about race and policing in America. Historical perspectives often point to slave patrols as one of the earliest forms of policing and the overlap between racist organizations (e.g., the Ku Klux Klan) and law enforcement bodies in the 19th and 20th centuries (17, 18). These historical accounts imply a routine practice of racist Whites seeking out posts in law enforcement to participate in the subjugation of non-Whites and the maintenance of racial hierarchy. Over the past decade, multiple reports have been issued documenting the entry, presence, and leadership of White supremacists in law enforcement agencies throughout the United States (19, 20). One report contends that “it is widely acknowledged that racist officers subsist within police departments around the country” and that explicit racism is “an especially harmful form of bias, which remains entrenched within law enforcement” (19). Even prominent reforms intended to redress racial bias in policing are predicated upon belief in the selection hypothesis. For example, a key assumption underlying implicit bias training for police officers is that bias in policing partially derives from negative stereotypes held by incoming officers (21). Perhaps more explicit, initiatives to diversify police forces are rooted in the logic that, “if you draw from a demographically different pool of recruits, one with overall lower levels of racial bias, then there should be less of a problem with racism on the force” (22). In short, the selection hypothesis appears axiomatic among activists, journalists, and policymakers in the United States as a component of systemic racism in law enforcement.

When reviewing decades of empirical social science research, however, the evidence in support of the selection hypothesis is surprisingly thin. Four veins of empirical literature bear on the selection hypothesis. One vein of research relies on surveys of law enforcement officers (13, 23–25), or administrative personnel records matched with voter files (26), to contrast officers’ social and political orientations with those held by civilian comparison groups. These studies typically find that officers are more right-leaning in their social views and political orientations than civilian comparison groups. Such studies, however, are unable to adjudicate between selection vs. police culture and occupational socialization (11, 27, 28) as sources of observed differences in attitudes between officers and civilians.

An alternative approach taken in a second vein of research is to survey cadets and junior officers—who have yet to undergo, or experienced only brief, occupational socialization—and compare their attitudes to civilian samples (29–31). These studies, however, typically rely on small samples collected within single jurisdictions and are conducted outside of the United States, leaving a conspicuous opening for replication in a nation where bias and excessive force in policing have been highly salient. A third vein of research investigates cadets’ and junior officers’ self-reported motives for joining the police force (32–34). While this literature finds that having power and authority are consistently important motives, studies in this vein do not compare the strength of these motives among officers to that observed among persons selecting civilian professions. Moreover, they do not ask questions that situate power and authority motives within the context of group hierarchies (i.e., having power and authority over ethno-racial minorities, immigrants, or LGBTQ people). A fourth vein of research explores interest in entering law enforcement among samples of late adolescents. Instead of studying people who have already selected to work in law enforcement—even if only recently (e.g., cadets)—this approach “rewinds” the process further back in time by studying interest in law enforcement among those at the career-selection stage of life (35–37). This approach is promising as a means of studying the genesis of selection early on in life; however, studies in this vein typically suffer from one conspicuous limitation: They do not measure attitudes toward lower status or stigmatized groups. One study within this vein of research comes close: Relying on samples of undergraduate students at a single American university, Sidanius and colleagues (16) found that students who viewed a career as a police officer as “very attractive” scored higher on social dominance orientation than peers viewing this career choice as “very unattractive.” However, while the social dominance scale used in this study captures general inegalitarian views (e.g., “This country would be better off if inferior groups stayed in their place”), it did not measure animus toward specific subordinated or stigmatized groups (e.g., Blacks, Latinos, immigrants, women, homosexuals).

Taken as a whole, these four veins of research inform us that individuals already working in law enforcement typically hold more right-leaning views than civilian comparison groups, though it remains unclear whether this is due to selection as opposed to occupational socialization. Moreover, individuals already working in law enforcement typically report that having power and authority were important motives for their career choice, though it is unknown if such motives reflect the desire to have power and authority over specific lower status or stigmatized groups. When evaluating studies of the career interests or intentions of late adolescents, we find this research comes up short either by not measuring group-related attitudes or only measuring general inegalitarian orientations (vs. antipathy toward specific groups). In sum, across these four veins of published research, there is a surprising scarcity of studies directly testing whether people drawn to a career in law enforcement hold more far-right and antipathetic views toward ethno-racial minorities, immigrants, women, homosexuals, drug-users, criminals, and other subordinated or stigmatized groups, than people drawn to alternative careers.

Our research builds on these past veins of scholarly work. First, we focus on the genesis of selection by examining the career intentions of American late adolescents (16 to 20 y) who are age-normatively entering adulthood and the career-selection stage of life. Unlike prior work using this approach, we leverage data containing a wealth of measures of views toward ethno-racial minorities, immigrants, women, homosexuals, drug-use, free speech, criminal justice, and the military. This allows us to test the following research question: Do late adolescents who gravitate toward a career in law enforcement hold more far-right, intolerant, and antiegalitarian social and political views than their peers leaning toward nonpolice careers or who have yet to formulate a career path? By asking and answering this question, we can shed vital light on whether longstanding bias in policing toward lower status and stigmatized groups is present as early as late-adolescence when Americans are prompted to contemplate possible careers and identify occupations they feel drawn toward.

Second, we utilize a dataset containing the reported career intentions and social and political views of over 13 million late adolescents collected across 44 y of time spanning every state and region of the United States. Using these data, we are able to overcome small sample issues affecting prior studies and precisely estimate parameters of interest. The immensity of these data also enable us to investigate and compare the relationship of career intentions to social and political views among important subgroups, such as Whites and non-Whites, and men and women. These subgroup analyses allow us to speak to reform initiatives aimed at enhancing ethno-racial and gender diversity in police forces as a means of mitigating bias and excessive force in law enforcement (38).

## Data and Methods

Our analysis uses the 1967–2010 waves of The Freshman Survey (TFS) conducted by the Cooperative Institutional Research Program. The TFS data, codebooks, and information about sampling and methods, are archived at the Higher Education Research Institute (https://heri.ucla.edu/). The TFS is administered to incoming first-year American college students during orientation before the start of classes and has a very high response rate (typically about 75% or higher). Every wave of the TFS contains 1) a question soliciting respondents’ intended career occupation and 2) modules of questions soliciting respondents’ social and political views, past and expected future behaviors, educational goals, and skill self-ratings. Given our focus on late adolescents at the career-selection stage of life, we restrict our analysis to respondents 16 to 20 y of age who comprise 95.2% of the total sample from 1967 to 2010. These 44 waves include N=13,203,930 respondents 16 to 20 y of age, an average of 300,089 respondents per wave, collected across 1,775 postsecondary institutions (46% at universities, 50% at four-year colleges, and 4% at two-year colleges) spanning 44 y. The institutions vary considerably in terms of size, geographic location, selectivity, student demographics, and public vs. private status. TFS respondents after 1981 reported their 5-digit home zip code and the data include respondents from all 50 states and covers nearly all counties (N=3,228) and zip codes (N=32,671).

The TFS is not designed to be a nationally representative sample of late-adolescent Americans; nevertheless, its unequaled immensity and wealth of measures provides an unrivaled opportunity to investigate the selection hypothesis, rendering it of high scientific value and able to offer a large contribution to our knowledge. When compared to young adults (i.e., those under 21 y) from a gold-standard representative survey like the American National Election Study (ANES), the late adolescents in the TFS do not drastically deviate on key dimensions from their peers sampled from the general population across decades from 1960 to 2010. As we show in *SI Appendix*, Fig. S1, the late adolescents in the TFS are slightly more white, less female (especially in the 1960s and 1970s), and drawn more from the Northeast and less from the South, than their peers appearing in the ANES. This corresponds roughly to what we expect the college-attending population to look like relative to the general population among this age cohort and across these decades. Perhaps most important, across all four decades for which we have data in the TFS and ANES, there are no appreciable differences in ideological self-placement (e.g., those who identify as politically “conservative”) between the TFS and the ANES. While it is possible that the late adolescents who appear in surveys like the ANES are more liberal than their peers who are harder to reach or less likely to consent to participate in social surveys, the comparisons in *SI Appendix*, Fig. S1 suggest that the late adolescents appearing in the TFS who selected to enter college are not markedly different in their liberal-conservative leanings from their peers dispersed among the general population.

### Using the TFS to Study Police Selection.

One concern worth addressing in using the TFS is that samples of first-year college students may differ from the people who enter and work in law enforcement. If few law enforcement officers have college degrees, or even enter college in the first place, it may limit the real-world applicability of information gained from studying first-year college students. Two considerations are relevant in guiding our use of the TFS to study the attitudes of young people gravitating toward law enforcement.

First, TFS respondents complete the survey during orientation before the start of classes; thus, while these individuals have selected to enter college, we observe their career intentions and social and political views before they are “treated” with some college education.

Second, the level of education of police officers in the United States drastically increased in the last quarter of the 20th century and remains high up to the present. A 2017 CSU-Fullerton survey of 958 police departments in the United States found that 51.8% of officers had at least a two-year degree, 30.2% had a four-year degree, and 5.4% had a graduate degree.^*^ Unfortunately, this 2017 survey did not measure the percentage of officers that enrolled in college but left prior to earning a degree. A 2016 Pew Research Center survey of N=7,917 law enforcement officers drawn from 54 municipal and county agencies (24) collected detailed information about the educational attainment of surveyed officers. This survey found that roughly 66% of officers had a two-year degree or higher and that another 27% reported having “some college but did not graduate.” Critically, this means that 93% of surveyed officers at least enrolled in college prior to joining the force. These estimates among surveys of officers are complemented by figures obtained from 1% random samples of raw Decennial Census and American Community Survey (ACS) data retrieved from IPUMS-USA.^†^ Among ACS or census takers whose reported occupation is police officer, weighted estimates indicate that 85.8% of officers in 2016, 80% in 2000, 62.2% in 1990, 60.9% in 1980, and 32.4% in 1970, had at least “some college” (*SI Appendix*, Table S1). The jump in educational attainment of officers between 1970 and 1980 has been attributed to commissioned reports on policing (e.g., the “Knapp Commission” and the National Advisory Commission on Criminal Justice and Standards) recommending higher educational standards for officers (e.g., requiring 2 y of college) and increased federal funding to enhance the education and training of officers (39, 40).

These figures greatly mitigate the concern that first-year college students and those entering law enforcement are starkly different subsets of the American population. On the contrary, as very large percentages of law enforcement officers enter college and have at least some college education, these figures suggest considerable overlap between first-year college students and those who work in law enforcement. Put simply, it is plausible that many TFS respondents intending a career in law enforcement go on to become police officers, rendering these large samples of late-adolescent Americans useful in testing the selection hypothesis. From 1980 onward, the plurality of surveyed officers in the census and ACS had either some college or a 2-y degree. Given this, we demonstrate in *SI Appendix* that our main findings largely hold, and in many instances become more pronounced, when focusing on subsets of respondents intending to be police officers that may more closely resemble this plurality of uniformed officers, such as those intending to be officers that attend a two-year (vs. four-year) institution or those self-reporting the intention to drop out of college before earning a degree (*SI Appendix*, Fig. S2). Additionally, we demonstrate that our findings hold when restricting the analysis to data collected between 1980 and 2010 when available estimates suggest the majority of law enforcement officers at least enrolled in college (*SI Appendix*, Fig. S3).

### Independent Variables.

TFS respondents were presented with roughly 47 distinct occupations and we constructed three binary variables each coded “1” for respondents who selected “law enforcement officer” as their likely career. The three different binary measures vary in terms of the comparison group: The first codes as “0” respondents who chose any other occupation or were undecided (labeled “all other”), the second codes as “0” respondents who chose any other nonmilitary civilian occupation or were undecided (labeled “all other civilian”),^‡^ and the third codes as “0” respondents who reported being undecided (labeled “undecided”), which is the modal category (13.6%) in the data, with all others excluded from the analysis. We analyze three measures with distinct comparison groups to ensure our findings do not significantly vary depending on to whom late adolescents intending a career in law enforcement are being compared. Across the 1967–2010 TFS, 108,400 respondents reported a likely career in law enforcement, with an average of N=2,464 (less than 1%) per annual wave (*SI Appendix*, Fig. S4). The number of intending officers in each annual TFS wave vastly exceeds the typical number of self-reported police officers contained in nationally representative samples of the American public (23, 25) or convenience samples of officers surveyed from cooperating agencies (13).

### Dependent Variables.

The dependent variables in our analysis include 33 questions appearing in various years of the TFS related to several issue areas: race relations, immigration and intercultural relations, women and gender roles, homosexuality and gay rights, drug use and control, free speech, criminal justice, and the military. These issue areas cover a broad range of stigmatized and subordinated groups and group-relevant policies in the United States and generally correspond to attitudes held by those higher in social dominance orientation (41). Our inclusion of views on drug use and control, free speech (e.g., protest and dissent), criminal justice, and the military can be viewed as validity checks, as we would expect those interested in a career in law enforcement to hold more punitive “law and order” views on drug-use, protest, and the treatment of criminals, and to support other protective services organizations.

Table 1 presents the items analyzed in each issue area (column 1), their question wording and question group (column 2), their label used in our analyses and figures (column 3), and whether they were reverse coded (column 4). Items in TFS questionnaires are members of thematic question groups with a group name^§^ and items from the same group share the same response options. All of the 33 outcomes we analyzed are ordinal, with either 3, 4, or 5 ordered response options. The outcomes listed in Table 1 come from 6 groups: the “VIEWS” group, denoted by (V) in column 2 of Table 1, soliciting social and political views (response options range from 1 = “Disagree strongly” to 4 = “Agree strongly”), the “ACT” group, denoted by (A), soliciting frequency of engagement in various activities in the past year (response options range from 1 = “Not at all” to 3 = “Frequently”), the “FUTACT” group, denoted by (F), soliciting the believed chances of engaging in various future behaviors (response options range from 1 = “No chance” to 4 = “Very good chance”), the “GOAL” group, denoted by (G), soliciting the personal importance of various goals associated with higher education (response options range from 1 = “Not important” to 4 = “Essential”), the “RATE” group, denoted by (R), soliciting self-ratings on skills and aptitudes compared to one’s peers (response options range from 1 = “Lowest 10%” to 5 = “Highest 10%”), and the “ADMITCONSIDER” group, denoted by (C), soliciting views on how much consideration college admissions officers should give to White, Black, Hispanic, Asian, and foreign applicants (response options range from 1 = “None” to 3 = “A lot”). From this last group, we created 4 items capturing the difference in preferred consideration given to Whites vs. each of the other 4 groups. We reverse scored 15 items (see column 4 of Table 1) so that higher values on all variables indicate holding more right-leaning positions. For example, for the question with the label *Future Socialize*, we reverse scored so that higher values indicate a lower reported likelihood of socializing with someone of another ethno-racial group; for the item *Equal Pay*, we reversed scored so that higher values indicate greater disagreement with pay and promotion parity between men and women. To ease interpretation and facilitate comparability in our analyses, we mean-standardize each of these 33 outcome measures by subtracting each outcome mean from the respondent-level outcome value and dividing that quantity by the outcome SD.

**Table 1.** Items analyzed from The Freshman Survey (TFS), 1967–2010

Issue area	Question wording (GROUP)	Label in figures	Reverse scored?
Race relations	“Racial discrimination is no longer a major problem in America” (V)	Discrimination	
	“Affirmative action in college admissions should be abolished” (V)	Abolish AA	
	“How much consideration should college admission officers give to Whites/Blacks/Hispanics/Asians?” (C)	Admit white–[Group]	
	“Busing is O.K. if it helps to achieve racial balance in the schools” (V)	Busing	✓
	“The federal government is not doing enough to promote school desegregation” (V)	Desegregation	✓
	In past year R–“Socialized with someone of another racial/ethnic group” (A)	Past socialize	✓
	Chances R will–“Have a roommate of different race/ethnicity” (F)	Roommate	✓
	Chances R will–“Socialize with someone of another racial/ethnic group” (F)	Future socialize	✓
Immigration & intercultural relations	“Undocumented immigrants should be denied access to public education” (V)	Deny education	
	“Children of undocumented immigrants should be denied access to public education” (V)	Deny children	
	“All official federal and state documents should be printed in English only” (V)	English only	
	Chances R will–“Participate in a study abroad program” (F)	Study abroad	✓
	Importance to R–“Improving my understanding of other countries and cultures” (G)	Understanding	✓
	R’s self-rating–“Ability to work cooperatively with diverse peoples” (R)	Cooperation	✓
	“How much consideration should college admission officers give to foreign students? (C)	Admit white–foreign	
Women & gender roles	“The activities of married women are best confined to the home and family” (V)	Stay home	
	“Women should receive the same salary and opportunities for advancement as men in comparable positions” (V)	Equal pay	✓
	“Just because a man thinks that a woman has ‘led him on’ does not entitle him to have sex with her” (V)	Sex entitled	✓
	“Abortion should be legal” (V)	Abortion	✓
Gay rights	“It is important to have laws prohibiting homosexual relationships” (V)	Prohibit relations	
	“Same-sex couples should have the right to legal marital status” (V)	Marriage	✓
	“Gays and lesbians should have the legal right to adopt a child” (V)	Adoption	✓
Drug use & control	“Marijuana should be legalized” (V)	Marijuana	✓
	“Employers should be allowed to require drug testing of employees or job applicants” (V)	Drug testing	
Free speech	“Colleges have the right to ban extreme speakers from campus” (V)	Ban speakers	
	“Student publications should be cleared by college officials” (V)	Publications	
	“Dissent is a critical component of the political process” (V)	Dissent	✓
	“Most college officials have been too lax in dealing with students protest on campus” (V)	Protest	
Criminal justice	“There is too much concern in the courts for the rights of criminals” (V)	Criminal rights	
	“The death penalty should be abolished” (V)	Death penalty	✓
Military	“Federal military spending should be increased” (V)	Spending	

None of the 33 questions we analyze were asked in every TFS wave, thus our analysis of each outcome relies on varying subsets of the data determined by the years the question was asked. *SI Appendix*, Fig. S5 illustrates which waves include each question. Some items were asked nearly every year (e.g. *Marijuana*) and others in just one year (e.g. *Deny Children*) (the average number of years a question was asked is 14). Our models have different sample sizes depending on the number of years in which the outcome item was asked, though we show in *SI Appendix*, Fig. S6 that there is no relationship across our analysis of these 33 outcomes between the sample size and observed effect sizes or the presence or absence of statistical significance.

### Models.

We use ordinary least squares models to regress each attitudinal outcome on a dummy indicator for likely career in law enforcement (labeled *Police Career*) using each reference category operationalization (“all other,” “all other civilian,” and “undecided”) across three separate models. Our 33 dependent variables were mean-standardized, so estimated coefficients for *Police Career* can be interpreted as the standardized shift in the outcome conditional on intending to be a law enforcement officer as opposed to a respective reference category. We control for confounders that are theoretically linked to selecting into a career in law enforcement and social and political views. These controls include gender, first-generation college student, ethno-racial self-identification, citizenship status, high school GPA, liberal-conservative ideological self-identification, parental education, parental income (calculated as within-year terciles for comparability across time), and parental employment in law enforcement. We pool the surveys and include a fixed-effect for survey year to control for time trends from external events. Our models include missing dummies for covariates with missing values; however, we show in *SI Appendix*, Figs. S7 and S8 that our results are substantively identical when estimated without controls (bivariate) or when list-wise deleting respondents with missing observations. All models use heteroskedastic-robust SEs clustered at the school level. See *SI Appendix*, Tables S2 and S3 for descriptive statistics on all variables we use in our analyses in addition to outcome means among comparison groups.

## Results

**Fig. 1.**
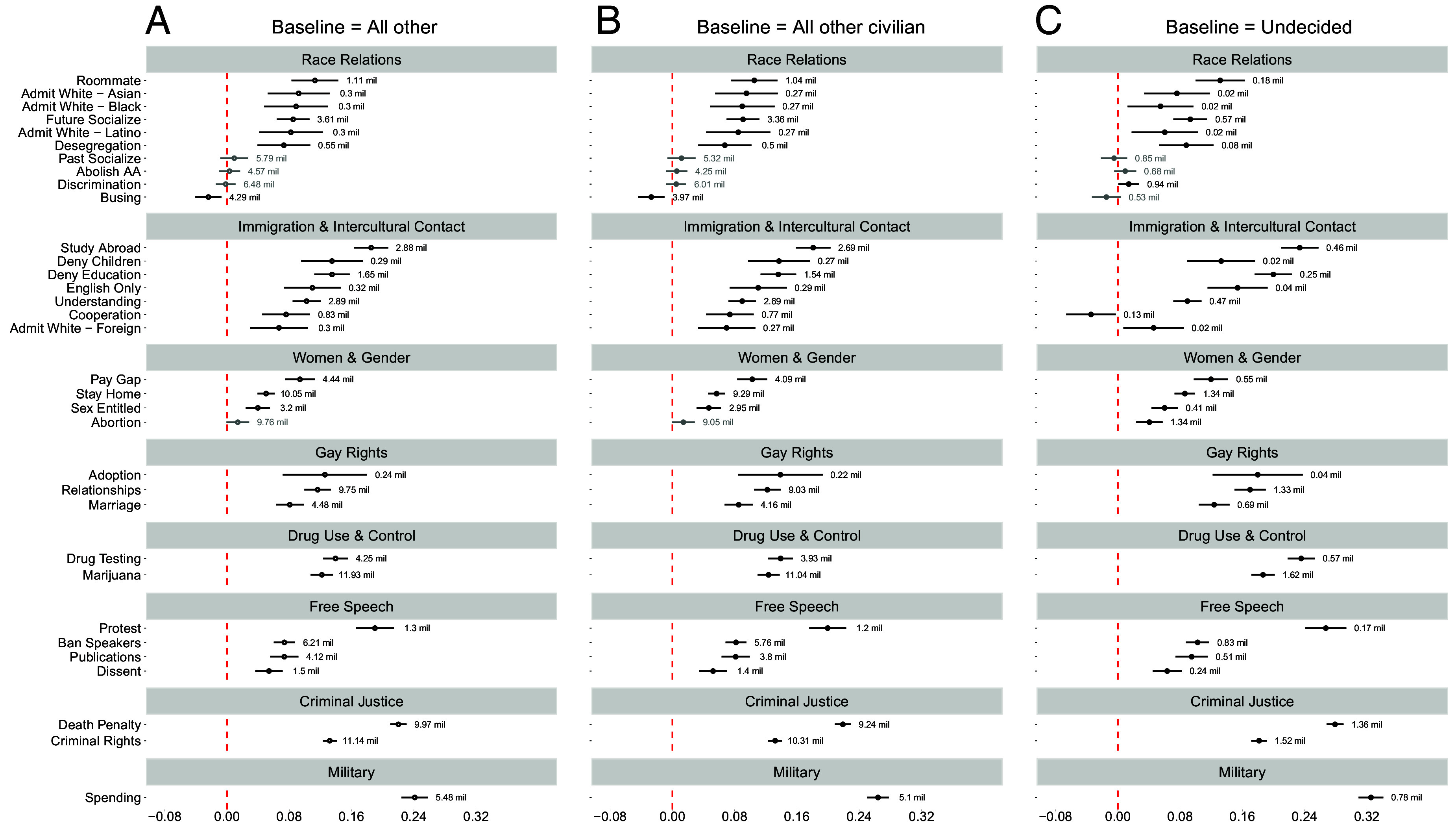
Late adolescents who intend to be police officers are more right-leaning on a number of attitudinal dimensions. The comparison group for Panel (*A*) are late adolescents who intend to pursue all other non-police careers. The comparison group for Panel (*B*) are late adolescents who intend to pursue all other civilian careers. The comparison group for Panel (*C*) are late adolescents who are undecided in their career intentions.

In Fig. 1, we display the standardized coefficient for likely career in law enforcement (*Police Career*) on each attitudinal outcome net of controls with 95% CIs. Sample sizes for each model are noted to the right of each estimate. We present results from models where respondents intending a career as law enforcement officers are compared to those intending all other careers (*A*), all other nonmilitary civilian careers (*B*), and the modal category who are “undecided” in their career intentions (*C*).

We begin with views on “Race Relations.” Across Panels (*A*–*C*), we find late adolescents gravitating toward a career as police officers are more likely than their peers to convey aversion to prospective interracial contact—reporting lower inclinations toward taking on roommates from different ethno-racial backgrounds or socializing with members of ethno-racial outgroups. These findings are statistically significant regardless of the comparison group used and extend to self-reported prior socializing with ethno-racial outgroups when restricting the comparison group to those intending all other civilian careers (*B*). Next, across the board (*A*–*C*), we find that intending police officers desire college admissions officers to consider White applicants over Black, Latino, and Asian applicants—a clear indicator of racial bias. With respect to views on race-related policies like desegregation, school busing, and affirmative action in college admissions, we uncover mixed findings. We find consistent and pronounced opposition to desegregation among intending police officers relative to their peers; however, support for abolishing affirmative action in college admissions is not significantly higher among intending police officers. Assuming views over college admissions serve as an arena for expressing racial animus, we find this animus more present among intending police officers when focusing on questions explicitly mentioning the admission of White students vis-a-vis various ethno-racial minority groups. The denial of racial discrimination is not consistently observed to a greater degree among intending law enforcement officers—we only observe a positive and significant coefficient for *Police Career* in Panel (*C*) when using undecided respondents as the comparison group. This weaker result may be due in part to the denial of racism representing a more recent means (e.g., following the rise of the Black Lives Matter movement) for prejudiced Americans to channel their racial animus (42). Finally, we observe a negative coefficient for *Police Career* across Panels (*A*–*C*) when analyzing views about school busing. This finding stands in contrast to the relationship of *Police Career* to most other outcomes in the “race relations” category and could potentially be due to ambiguity in question wording and potential variation in respondents’ interpretation of the meaning of “achieve racial balance.” Aside from this outlying finding, the results for race relations indicate an overall pattern where late adolescents gravitating toward a career in law enforcement evince more contact-averse, White-favoring, and right-leaning, orientations than their peers.

Turning to views on “Immigration & Intercultural Relations,” the results are striking: Late-adolescent Americans in the TFS interested in a career in law enforcement convey significantly more anti-immigrant views than their peers—supporting the denial of access to public education to undocumented immigrants and their children. When it comes to cultural concessions to immigrants in the form of accommodating nonnative speakers, those intending to be law enforcement officers are significantly more supportive of an “Official English” policy of making government documents available in English-only. With respect to intercultural relations, those gravitating toward a career in law enforcement convey significantly less interest in broadening their cultural horizons by studying abroad or attending college to improve their understanding of different countries and cultures. Moreover, we find evidence in Panels (*A* and *B*) that intending police officers view themselves as less competent in navigating interaction with diverse peoples than their peers. Finally, intending officers evince bias in the form of favoring the college admission of Whites over foreign students. In sum, young Americans starting college that plan to enter into law enforcement are more supportive of restrictive immigration policies, and less interested in expanding their cultural horizons, than their peers.

Views concerning “Women & Gender” and “Gay Rights” follow similar patterns. Respondents intending a career as law enforcement officers hold more hostile sexist views than their peers and are less supportive of women’s reproductive rights when the comparison group is late adolescents who have not decided their career. These individuals are more likely than their peers to believe that women should not receive the same salary and opportunities for advancement as men in the same positions and that the activities of married women are best confined to the home and family. Strikingly, those intending a career in law enforcement express significantly more sex entitlement than their peers—being more likely to think that men are entitled to sex with women if the women in question “lead them on.” Turning our attention to “Gay Rights,” we find that young Americans entering college planning on becoming police officers are more likely than their peers to oppose laws allowing gay couples in the United States to engage in sexual relationships, adopt children, or get married. Taken together, we find robust evidence that TFS respondents gravitating toward a career in law enforcement have more antiegalitarian and right-leaning views than their peers on gender, sexual relations, and gay rights.

Finally, we examine the relationship between intending a career in law enforcement and support for “law and order” stances on drug use and control, free speech, criminal justice, and military funding. First, those intending a career in law enforcement are substantially more opposed to the legalization of marijuana and more supportive of drug test requirements for employees. Next, intending police officers are less supportive of free speech—registering stronger opposition to college protesters, support for banning extreme speakers, support for oversight of student publications by college administrators, and overall opposition to dissent in the political process. The relationship between the intention to be a police officer and belief that colleges have been “too lax” in dealing with college protesters (i.e., a call for order on college campuses) is particularly strong. Prior to even joining police academy or being socialized into police forces, those leaning toward pursuing a career in law enforcement evince an aversion to dissent and inclination toward using institutional authority to curtail free speech and protest. These findings have important implications for the prospect of democratic integrity in the United States. Oppressed groups often use protest as a tool for elevating public awareness of their experienced prejudice and discrimination (43); however, we know that state repression is a common response to protest and that police forces are routinely deployed to suppress protest (44, 45). The findings presented here suggest that the people who select into law enforcement tend to hold more antipathetic views toward lower status groups likely to engage in protest and may be somewhat more amenable to the suppression of the exercise of free speech and assembly by these groups. Finally, we observe pronounced positive relationships for (lack of) concern for the rights of criminals and support for the death penalty and increased military spending (i.e. international “law and order”).

We have described the association between *Police Career* and our 33 outcomes largely in terms of sign and statistical significance. To give a sense of the size and substantive significance of the results in Fig. 1, we 1) benchmark them to those observed for liberal-conservative ideological self-identification, 2) place them in the context of the size of effects reported in other studies using the TFS, and 3) place them in the context of the size of observed differences in attitudes between law enforcement officers and the general public in published studies with available replication data. First, we benchmark the *Police Career* coefficients in Fig. 1, Panel (*A*), by comparing them to the size of the coefficients for liberal-conservative political ideology from the same models (*SI Appendix*, Table S4). We use ideology as a benchmark because prior research establishes it as highly prognostic of group-related attitudes and antiegalitarian beliefs (13, 41). Across 33 models, we find that the standardized *Police Career* coefficient is between 4 and 145% of the min-max standardized ideology coefficient for statistically significant and positive outcome tests, with a mean of 20% (median 15%). An average effect of *Police Career* that is two-tenths of the effect of ideology is substantively significant given the established strength of association of ideology with inegalitarian attitudes. Second, we compare the standardized coefficients for *Police Career* from Fig. 1, Panel (*A*), to standardized coefficients for key predictors in other published studies using the TFS (*SI Appendix*, Table S5). We find that the positive and statistically significant effect sizes we observe for the *Police Career* variable across our outcomes in Fig. 1 Panel (*A*) (range = 0.04 to 0.24, mean = 0.11) are comparable to effect sizes reported (range = 0.062 to 0.17, mean = 0.10) for various predictors of an array of social and political attitudes in these published studies. Third, we compare the standardized coefficients for *Police Career* from Fig. 1, Panel (*A*), to standardized coefficients capturing the differences in social and political attitudes between law enforcement officers and the general public in two recently published studies (23, 25) (*SI Appendix*, Fig. S9). We find that the average standardized effect of intending to be a law enforcement officer in our analysis represents 41 to 50% of the average standardized size of differences between law enforcement officers and the public in these two studies. Given that our estimates are more likely to characterize selection effects (vs. selection + occupational socialization), an extrapolation that could be made from these coefficient comparisons is that selection may explain a substantively meaningful portion of the difference in right-leaning attitudes between adult law enforcement officers and the general public.

The findings presented in Fig. 1 are robust to a series of checks, including the addition of sample weights (*SI Appendix*, Fig. S10), the use of ordinal regression models (*SI Appendix*, Table S6), and the use of composite scaled outcomes by issue area as dependent variables (*SI Appendix*, Figs. S11 and S12). Our results are also robust to the inclusion of potential contextual confounders (e.g., county-level population density and state-level Republican vote share from the most recent prior presidential election), state fixed effects, and SEs clustered by both school and year (*SI Appendix*, Fig. S13). Our results are also robust to dichotomizing the outcomes based on agreement (disagreement) with the conservative (liberal) position (*SI Appendix*, Fig. S14) and dichotomizing the outcomes where “1” is equal to the maximum value of the ordinal scales across outcomes (*SI Appendix*, Fig. S15). Finally, while readers might be concerned about multiple hypothesis testing with 33 different outcomes, we find the same number of estimated coefficients (29 out of 33) for *Police Career* are statistically significant when using a Bonferroni-correction adjusted *P*-value (P=0.0015) as are significant in Fig. 1 when using a *P*-value of 0.05.

### Heterogeneity by Time.

Given that our data span nearly four and half decades (i.e., 1967–2010), it is important to address and dispel two potential interrelated forms of “ahistoricism” (46) in our analysis. The first applicable form of ahistoricism involves “slicing into history” in an atheoretical fashion driven by data availability. Borrowing language from Isaac and Griffin (46), the police selection hypothesis alludes to a “macrosociological process” (873) that could change over time. While the 1967–2010 time period was determined by the availability of TFS data, existing theory and evidence do not lead us to expect drastic variation in the hypothesized process of police selection across this 44 y period. It is undeniable that this time period was dense with events, movements, and societal changes related to race, immigration, gender, sexuality, and drug use, to name a few. One may also point to the changes in law enforcement that occurred in response to recommended reforms emerging from government-commissioned reports in the 1960s and 1970s (39, 40). Nevertheless, existing scholarship contends that, despite reforms to law enforcement (e.g., diversity-in-hiring initiatives), police organizations remain gendered and racialized with respect to their inner workings (e.g., guiding logics, policies, and practices), with the result being that police work continues to reproduce various social inequalities (28). Moreover, there is evidence that bias in law enforcement against the various subordinated groups in our analysis was present throughout our study period (1–3, 5, 7). This work implies that the theorized status of law enforcement agencies as “hierarchy-enhancing” institutions remained throughout our study period, suggesting the sustained applicability of the police selection hypothesis.

A second and related applicable form of ahistoricism involves the presumption that coefficients of interest are constant over the entire time period under investigation. To address this, we tested for heterogeneity in the relationship between *Police Career* and our outcomes by year. We ran two sets of models, one bivariate and the other fully specified, for each model in each year and plotted the coefficients in *SI Appendix*, Fig. S16. We show that, while the relationship between *Police Career* and our attitudinal outcomes does vary modestly year-to-year, the pattern of results is remarkably stable and consistent over time. This is noteworthy given the macrosocietal shifts happening over the time period that these surveys were in the field.

### Heterogeneity by Race and Gender.

One proposed means of reducing bias and excessive force in policing is to diversify police forces, which have largely been dominated by Anglo-White males (26). Recent research finds that police use of lethal force is lower in departments headed by non-White leaders (47) and that non-White and female officers are less likely to use force than their White and male counterparts (38). Accompanying this, prior research finds starker differences in antiegalitarian views between police officers and civilians when focusing on Whites (13) and that holding antiegalitarian views is most strongly correlated with use-of-force when analyzing White officers (48). Taken together, these findings suggest that, to the extent negative attitudes toward lower status groups are visible among those selecting into law enforcement, this relationship should be most present among White and male late adolescents relative to their non-White and female peers who are members of lower status and subjugated groups. This expectation aligns with the rationale behind efforts to recruit more non-White and female officers (38), as the former are presumed to harbor less bias against non-Whites, the latter are presumed to harbor less bias against women (e.g. in sexual assault or other gender-related cases), and both groups are believed to be less inclined to use unnecessary force against civilians.

Opposing these expectations, however, is the possibility that the police selection hypothesis applies across race and gender, such that non-Whites and females gravitating toward police work are more right-leaning in their social and political views than their fellow group members planning other lines of work. This would be consistent with a central assertion of person-organization congruence theory (14), which is that people gravitate toward organizations to which they detect compatibility between their individual inclinations, motivations, and worldviews and the societal functions of the organization with respect to the attenuation or enhancement of social hierarchy. As such, it may be the case that non-White and female young adults that select into becoming police officers start out with more hostile views toward lower status groups than their peer group members gravitating toward nonpolice career tracks. This expectation is supported by research finding that non-White young adults holding more punitive attitudes toward criminals are more likely to express a desire to work as a law enforcement officer (49). This expectation is also supported by research finding that White and Black and male and female police officers report similar power and authority oriented motivations for entering law enforcement (34). To dig into these competing possibilities, we reanalyze the results in Fig. 1 by re-estimating our models among subgroups defined by the reported race/ethnicity or gender of TFS respondents.

**Fig. 2.**
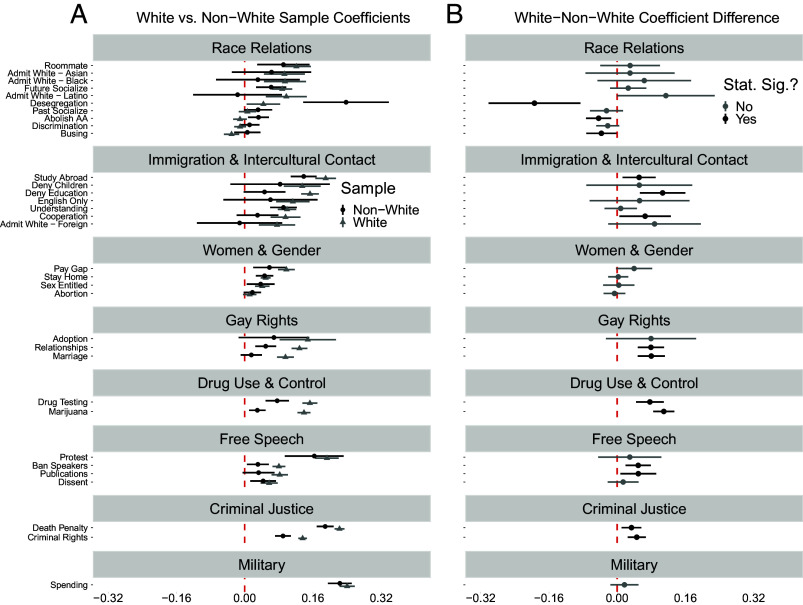
Association between police officer intention and conservative attitudes conditional on race among late adolescents. Panel (*A*) characterizes the association between police officer intention and the different outcomes by race (Non-White, White, denoted by color). Panel (*B*) characterizes the difference in the police officer intention coefficient for Whites and Non-Whites (White–Non-White). Higher values on Panel (*B*) mean the association between police officer intention and conservative attitudes is stronger for White late adolescents relative to Non-White late adolescents.

Panel (*A*) of Fig. 2 displays covariate-adjusted coefficients for *Police Career* (baseline = all other) for each outcome with 95% CIs conditional on whether the respondent identifies as non-White (e.g., Black, Latino, Asian, American Indian) or White. Fig. 2, Panel (*B*), presents the differences in the coefficients plotted in Panel (*A*) between White and non-White respondents for each outcome. Out of 33 social and political views analyzed, we observe statistically significant differences between Whites and non-Whites (*B*) in the relationship between *Police Career* and attitudes in 14 instances—less than half (42%) of the time. Of these 14 instances in Panel (*B*) where significant differences in the relationships under investigation are observed, 3 of these instances pertain to views on race relations and involve *negative* coefficient differences—indicating that the size of the relationship between *Police Career* and holding more racially conservative views than their peers is *larger* for non-Whites than Whites. Indeed, for 3 of the 10 race relations outcomes (“Desegregation,” “Abolish AA,” and “Busing”) the coefficient for non-Whites is statistically larger than the coefficient for Whites (at least P<0.05). And, for 1 of these (“Abolish AA”), the coefficient for non-Whites in Panel (*A*) is positive and statistically significant while the corresponding coefficient for Whites is negative and insignificant—indicating that non-Whites who intend to be law enforcement officers oppose affirmative action more than their non-White peers likely pursuing other careers while the same difference in views on affirmative action by career intentions is not present among Whites. Of the remaining 11 instances in Panel (*B*) where there is a positively signed statistically significant coefficient difference between Whites and non-Whites, 9 of these 11 instances still involve a positive and statistically significant coefficient for *Police Career* for non-Whites in Panel (*A*)—indicating that non-Whites who planned to be police officers still held more right-leaning views on immigration, gay rights, and “law and order” issues than their non-White peers planning other career tracks.

In short, the results in Fig. 1 are not solely driven by White Americans; to the contrary, we observe support for the selection hypothesis for both White and non-White late-adolescent American college freshmen gravitating toward a career in law enforcement. Non-White young Americans who viewed it as likely they will become law enforcement officers held more right-leaning views on race relations, immigration and intercultural contact, women and gender roles, homosexuality and gay rights, drug use and control, free speech, and criminal justice, than their non-White counterparts interested in pursuing other occupations. While the magnitude of these relationships are somewhat smaller for non-Whites nearly half of the time, they are nonetheless largely still present in these instances. Taken together, across over 40 y of data, these findings suggest that the non-White late-adolescent Americans planning to enter law enforcement were predisposed relative to their non-White peers toward the maintenance or enhancement of social hierarchies, as evinced by their relative opposition to policies intended to ameliorate racial inequality (e.g., desegregation, busing, affirmative action), endorsement of punitive immigration policy and lack of interest in gaining cultural competence, support for traditional gender roles and gender inequality, opposition to homosexuality and gay rights, punitive orientations toward drug users and criminals, and support for suppressing protest, controversial speech, and dissent.

**Fig. 3.**
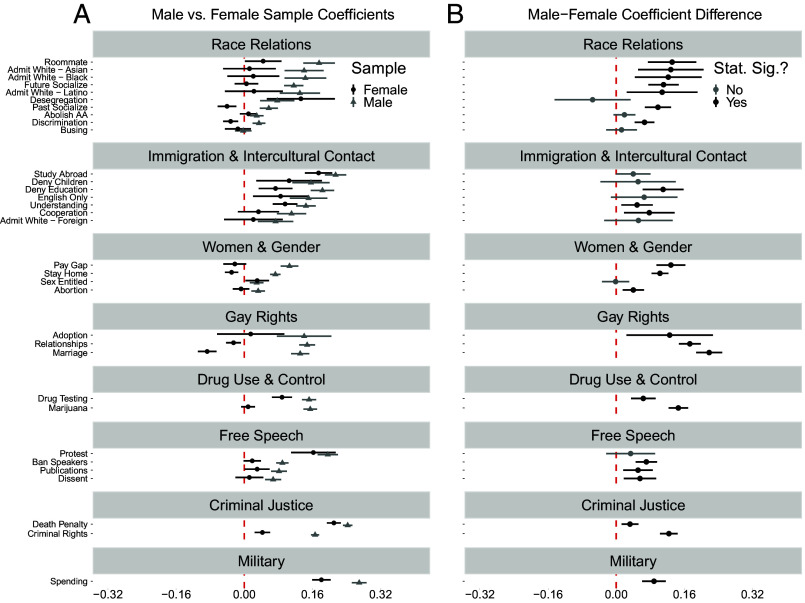
Association between police officer intention and conservative attitudes conditional on gender among late adolescents. Panel (*A*) characterizes the association between police officer intention and the different outcomes by gender (Female, Male, denoted by color). Panel (*B*) characterizes the difference in the police officer intention coefficient for Male and Female (Male–Female). Higher values on Panel (*B*) mean the association between police officer intention and conservative attitudes is stronger for late adolescents who are male relative to late adolescents who are female.

Turning to our analyses by gender, Fig. 3, Panel (*A*) displays covariate-adjusted coefficients for *Police Career* (baseline = all other) on each outcome with 95% CIs conditional on whether the respondent identified as a female or male. Fig. 3, Panel (*B*) presents the differences in the coefficients plotted in Panel (*A*) between male and female respondents for each outcome. Out of 33 analyzed views, we observe statistically significant differences between males and females in the relationship between *Police Career* and attitudes in 24 instances—roughly 73% of the time. Importantly, in each of these instances, the coefficient for *Police Career* is larger among male than female respondents; what is more, in 16 of these 24 instances, the coefficient for *Police Career* among female respondents in Panel (*A*) is either statistically insignificant (e.g, “Future Socialize”) or significant but *negatively* signed (e.g., “Marriage”). Focusing specifically on the 10 items concerning race relations, we observe significant gender differences in coefficients in Panel (*B*) for 7 of the outcomes and for 6 out of these 7 outcomes the results in Panel (*A*) indicate that women who intend a career in law enforcement either hold views similar to, or significantly more racially liberal than their female counterparts intending other careers. In contrast to the results in Fig. 2, the findings in Fig. 3 suggest that hiring more female officers may alleviate racially disparate outcomes in policing deriving from officer bias. The late-adolescent freshman women tracking themselves toward law enforcement hold racial views comparable to, or more liberal than, their female peers whereas their male counterparts intending to be police officers are consistently more racially conservative than their male peers interested in nonpolice occupations.

One area where women interested in law enforcement appear as distinct among their gender as their male counterparts intending a law enforcement career is in the issue domain of immigration and intercultural relations. We observe positive and significant coefficients for *Police Career* among female respondents in 5 out of 7 instances, and in the one instance in Panel (*B*) where there is a significant gender difference in the *Police Career* coefficients, we observe a positive and significant coefficient for women in Panel (*A*) (“Deny Education”). Thus, while freshman women tracking themselves toward law enforcement do not appear distinctly racially conservative relative to their female peers, they are distinctly supportive of restrictive immigration policies, averse to intercultural contact, and uninterested in gaining cultural competence. Several additional forms of heterogeneity by gender are worthy of note. First, female respondents intending a career as a law enforcement officer exhibit lower levels of sexism and more pro-gay attitudes than their female peers interested in other occupations. The opposite is true for male respondents in these issue domains. Finally, when it comes to various “law and order” views linked to drug use, free speech, criminal justice, and the military, we see that the coefficients for *Police Career* for women, even when they significantly differ from men (*B*), are nonetheless positive and statistically significant in Panel (*A*) (e.g., “Drug Testing,” “Publications,” “Death Penalty,” “Criminal Rights,” and “Spending”).

In sum, across 44 y of data, the findings presented here suggest that, while subscribing to more conservative “law and order” views than their female peers, late-adolescent women entering college planning to work in law enforcement were not distinctly hostile toward (nonimmigrant) ethnic or racial minorities, leaned feminist in their views toward women and gender, and were decidedly pro-gay. These findings stand in contrast to male late-adolescent Americans interested in a career in law enforcement, as this subgroup of males leaned more racially conservative, sexist, and antigay than their male counterparts. Where male and female late adolescents interested in law enforcement seemingly converge is in their aversion to immigration and intercultural contact, punitive orientations toward drug users and criminals, and support for suppressing protest. In short, female young adults enrolled in college gravitating toward law enforcement appear predisposed relative to their same-gender peers toward maintaining social hierarchy for several groups (drug users, criminals, protesters and dissidents) while simultaneously predisposed toward attenuating such hierarchy with respect to ethno-racial minorities, women, and homosexuals.

**Fig. 4.**
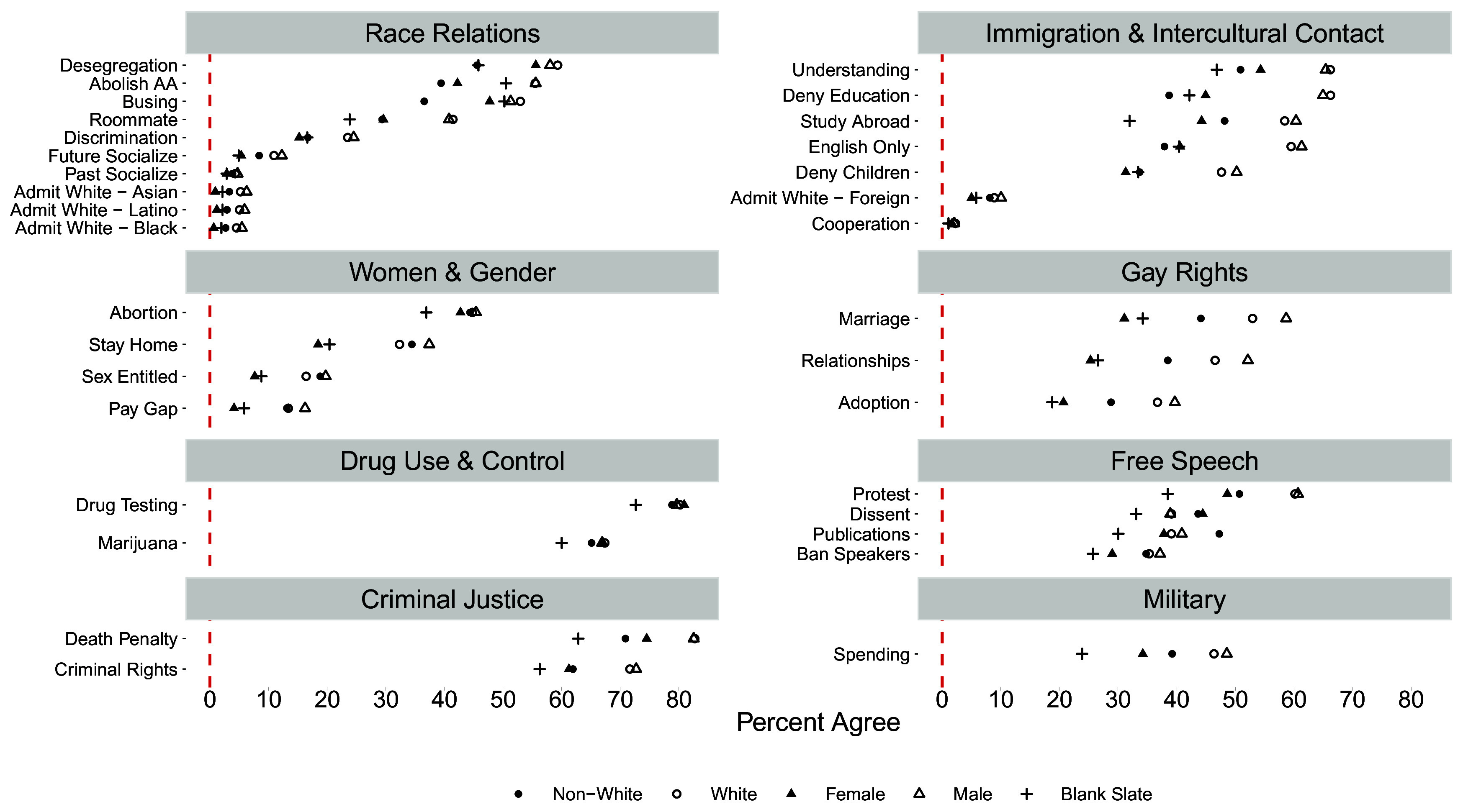
Proportion of each subgroup of intending law enforcement officers (and “blank slate” undecideds) holding right-leaning view.

While the results presented in Figs. 2 and 3 provide information about the presence and magnitude of differences in views between those intending to be law enforcement officers vs. other occupations among White, non-White, male, and female late-adolescent respondents, they do not provide an overall picture of the comparative proportions of each subgroup of intending police officers reporting right-leaning positions across the 33 solicited views. Providing such an overall picture would allow us to compare the levels of right-leaning views between these four subgroups of intending officers, which would give us a sense of which group falls the furthest to the right or left. Fig. 4 depicts the proportion of White, non-White, male, and female late-adolescent TFS respondents intending to be law enforcement officers reporting right-leaning positions on each view. For example, in the domain of race relations, we plot the proportion that agreed with abolishing affirmative action, agreed that racial discrimination is no longer a problem in America, opposed school desegregation, reported little-to-no past or future interracial contact (e.g., cohabitation or socializing), and favored Whites over each non-White group in college admissions. As an additional example, in the domain of free speech, we plot the proportion that agreed with banning extreme speakers from campus, agreed that colleges were being “too lax” in dealing with protests on campus, agreed with college officials censoring student publications, and disagreed with the notion that dissent is a critical component of the political process.

For ease of interpretation, we arrayed the outcomes on the y-axis of Fig. 4 from the highest to lowest proportion of right-leaning views. To compare the proportion of right-leaning views among these four subgroups to a general peer group, we also plot for each outcome the proportion of career-undecided respondents (i.e., the modal category first-year American college student) reporting right-leaning positions.

Beginning with race relations, we observe consistently higher proportions of conservative responses among White or male intending officers relative to non-White or female intending officers. Focusing specifically on policy-related items (e.g., “Desegregation,” “Abolish AA,” and “Busing”), we find that non-White intending police officers have the lowest relative proportion of right-leaning views and the differences in proportions between Whites and non-Whites are largest. When it comes to explicitly favoring Whites over non-Anglo groups in college admissions, we see overall the lowest levels of bias; yet, such explicit bias is more prevalent among male or White intending officers. Turning to immigration and intercultural contact, the picture is the same: We observe consistently higher proportions of anti-immigrant and contact-avoidant responses among White or male, than non-White or female, intending officers. This said, one thing that is notable within this attitude domain is that lack of interest in intercultural contact (“Study Abroad”) or gaining cultural competence (“Understanding”) are both higher among non-White and female intending officers than their career-undecided peers. Turning to views on women and gender, we observe the lowest proportion of gender inegalitarian views among female intending officers, though the four subgroups tend to cluster together in terms of opposing legal abortion. Similarly, we see the lowest proportion of antigay views among female intending officers. Finally, for views on drug use, free speech, criminal justice, and the military, we observe consistently higher proportions of “law and order” oriented right-leaning views among each subgroup than their career-undecided peers.

In sum, among respondents planning to pursue a career in law enforcement, White and male intending officers harbor the most right-leaning attitudes. With the exception of Non-White intending cops, who are more liberal than their peers on race-relations, it is women who, nearly across the board, hold the least conservative attitudes and look most similar to their “undecided” peers. This offers suggestive evidence that, by holding the lowest levels of potentially problematic attitudes, women who select into policing are less likely to engage in biased behavior toward marginalized groups (immigrants aside) once in uniform.

## Conclusion

This article makes a vital contribution to the scientific study of law enforcement, political psychology, and public opinion by offering a much needed test of the selection hypothesis. Using survey data on 13 million Americans spanning 44 y, we offer the most comprehensive picture to date of the social and political views of late adolescents gravitating toward a career in law enforcement. Compared to peers planning to enter other occupations or who have not formulated a career path, young Americans planning a law enforcement career harbor more antipathetic and inegalitarian views toward ethno-racial minorities, immigrants, women, homosexuals, drug-users, dissidents, and criminals. Prior research demonstrates that law enforcement officers are right-leaning, authoritarian, antiegalitarian, and biased against lower-status social groups and that these orientations are consequential in terms of their behavior on the job (48, 50). Our findings strongly suggest that these characteristics of police forces are partly the product of selection.

In light of recent leading research on ethno-racial and gender diversity in law enforcement (38), we explored whether our results were driven by White or male young Americans. Critically, we uncover evidence for the selection hypothesis among non-White Americans, as those gravitating toward a career in law enforcement consistently held more right-leaning social and political views than their non-White peers intending other occupations. This said, while more right-leaning than their non-White peers, non-Whites who plan to enter law enforcement are overall less right-leaning than their White peers aiming to become police officers. We observed the most significant attenuation in selection among women planning to enter law enforcement, as they held views similar to, and on some issues—such as women’s and gay rights—significantly more liberal than, their female peers intending other occupations. Furthermore, in several cases where we observed a significant relationship between career intentions and right-leaning views among females, this relationship was smaller for females than males—suggesting stronger selection among males. Finally, while in some instances females gravitating toward law enforcement were more right-leaning than females leaning toward other occupations, females who plan to enter law enforcement were overall less right-leaning than their male peers who aimed to become cops.

Our research suggests a focus of police reform efforts should be tests and filters implemented during officer recruitment. While hiring more non-White and female cadets may yield officers with less antipathy toward some societal groups, our findings suggest such individuals may still possess negative orientations toward many other groups they could encounter in the line-of-duty. According to the International Association of Police Chiefs, law enforcement hiring typically involves testing for psychological suitability, emotional stability, and traits associated with successful field performance. Consistent with prescriptions by police reform advocates (51), recruitment processes could also screen for psychological skills needed to counteract bias (52). The state of California’s Peace Officer Standards and Training (POST) manual, for example, requires psychologists to screen recruits for explicit and implicit biases toward groups based on their race or ethnicity, gender, religion, disability status and sexual orientation. Further, POST criteria include psychological screening to assess dimensions related to cultural competency like adaptability, empathy, and tolerance (53). Finally, while these screening criteria could filter out candidates most predisposed to engage in biased behaviors, our findings do not speak to what happens once new recruits are socialized by peer officers. Future research should investigate the extent to which antiegalitarian attitudes change throughout a police officer’s tenure, the comparative magnitude of these changes across officer subgroups, and interventions that might disrupt any negative socialization effects experienced in the field.

## Supplementary Material

Appendix 01 (PDF)

## Data Availability

All raw study data in addition to the data and code to reproduce all figures and tables in the main text and *SI Appendix* can be accessed in the article, the *SI Appendix*, and the following Open Science Framework repository: https://osf.io/tzfb9/ (54).

## Footnotes

^*^https://www.nu.edu/blog/law-enforcement-education/.

^†^https://usa.ipums.org/usa/.

^‡^We include this version of the comparison group because the literature identifies the military as a prime “hierarchy enhancing” institution (14).

^§^See the TFS master codebook here: https://ucla.app.box.com/v/TFS-Trends-Survey-Items-xls.
