# Supplementary material for: Late adolescents entering college intending a career as police officers hold more right-leaning views than their peers
Source: Proc Natl Acad Sci U S A. 2025 Aug 6;122(32):e2500220122. doi: 10.1073/pnas.2500220122 (PMC12358885; doi:10.1073/pnas.2500220122)
Supplement: Supplementary file 1 — Appendix 01 (PDF) [file pnas.2500220122.sapp.pdf]

Supporting Information for

Tyler T. Reny, Marcel F. Roman, Benjamin J. Newman, and David O. Sears

Corresponding Author Benjamin J. Newman.
E-mail: bnewman@ucr.edu

This PDF file includes:

Figs. S1 to S16
Tables S1 to S6
SI References

1. Representativeness of TFS

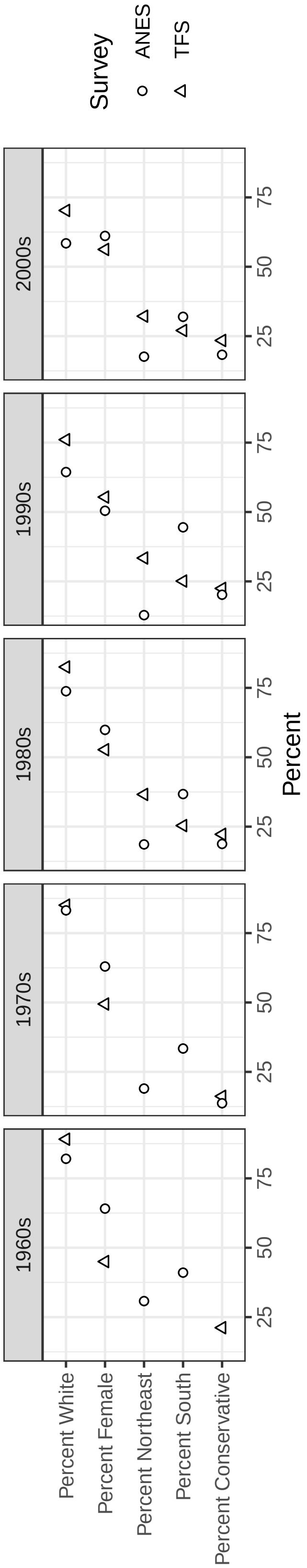

Fig. S1. Note: Demographic comparison of those under 21 in TFS relative to the cumulative American National Election Study (ANES) grouped by decade.

2. Police Officer Education Statistics

Year	No College	Any College	Some College	Associates Degree	Bachelors Degree	Higher
1970	67.60	32.40	24.17	6.61	1.62	0.00
1980	39.13	60.87	36.98	19.31	4.58	0.00
1990	37.76	62.24	34.58	12.44	13.03	0.43
2000	20.04	79.96	39.98	14.66	21.94	0.56
2006	19.07	80.93	33.40	17.38	25.75	0.61
2011	15.90	84.10	35.69	16.98	27.09	0.50
2016	14.17	85.83	31.80	16.63	30.78	0.93
2021	14.93	85.07	29.66	16.93	30.83	1.11
2022	14.68	85.32	29.68	16.13	32.22	0.60

Table S1. Officer Education from IPUMS Census and ACS Micro-Data 1970-2010 (weighted means)

3. Heterogeneity by College Student

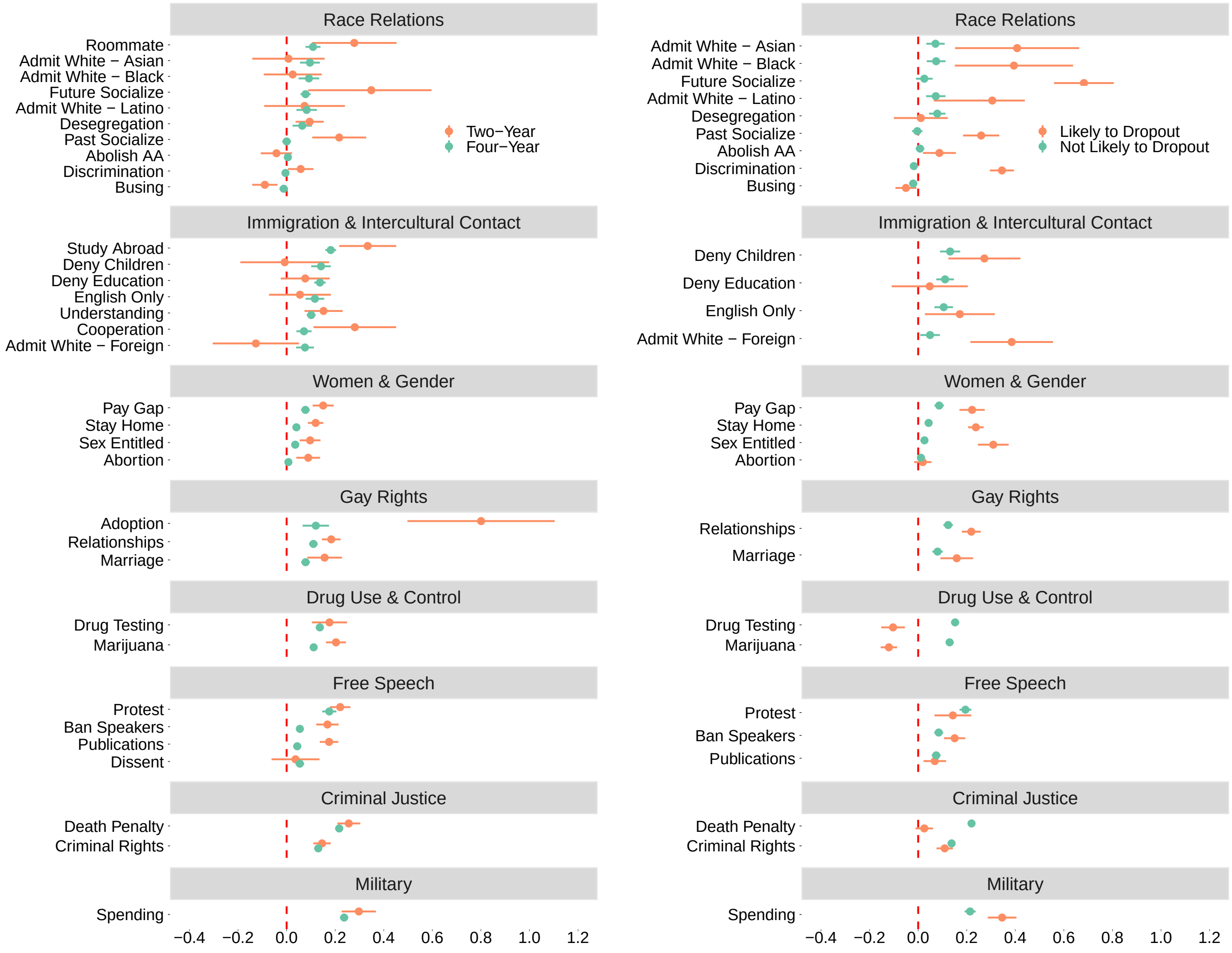

Fig. S2. Re-analysis of results in Figure 1, Panel A, focusing on intending law enforcement officers enrolled at two-year vs. four-year institutions (left figure) or intending law enforcement officers who indicate that they are likely ("some chance" or "very good chance") or not likely to dropout of college (right figure). This figure presents the results from four ancillary regression models. For these four models, the respondents in the reference group coded "0" on *Police Career* remain the same (0 = All Other Career intentions). The respondents coded "1" on *Police Career* are altered to include those intending to be a law enforcement officer that are attending a two-year college (left figure, orange colored estimates), four-year college (left figure, green colored estimates), likely to dropout of college (right figure, orange colored estimates), or not likely to dropout of college (right figure, green colored estimates). All coefficients presented are mean standardized. Missing outcomes across subset exercises (e.g. no "adoption" outcome for Gay Rights outcome category on right-panel when re-analyzing results subsetting by dropout likelihood) are due to missing data on both the subset and outcome variable. 95% CIs displayed.

4. Heterogeneity by Time

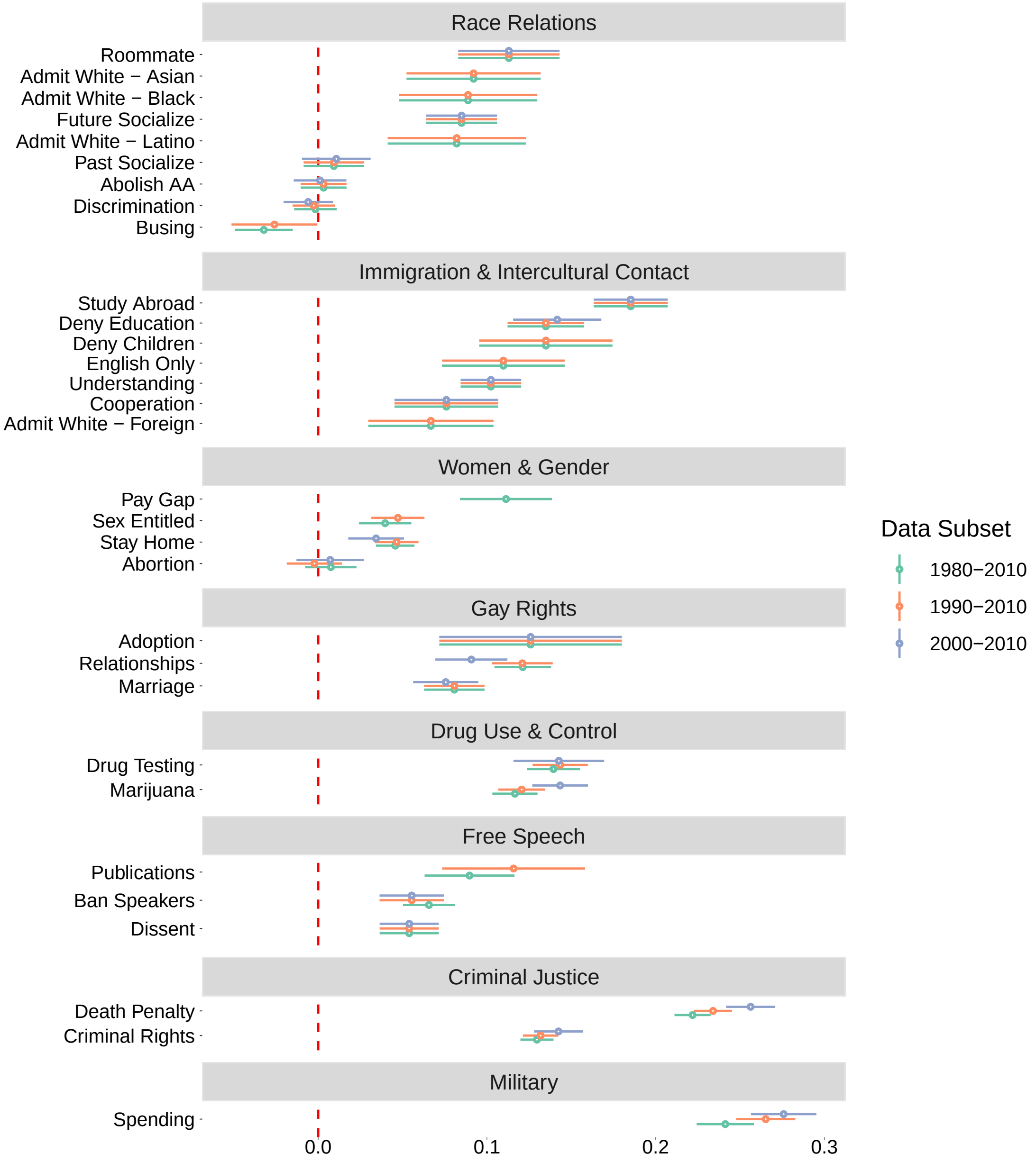

Fig. S3. Modeling results using pooled data 1980-2010, 1990-2010, and 2000-2010. Missing coefficients are the product of certain outcome items not being asked during particular time periods (e.g. “Publications” outcome under the Free Speech outcome category is missing during 2000-2010 because it was not asked on the TFS during that time period). All coefficients presented are mean standardized. 95% CIs displayed.

5. Self-Reported Police Career Intention

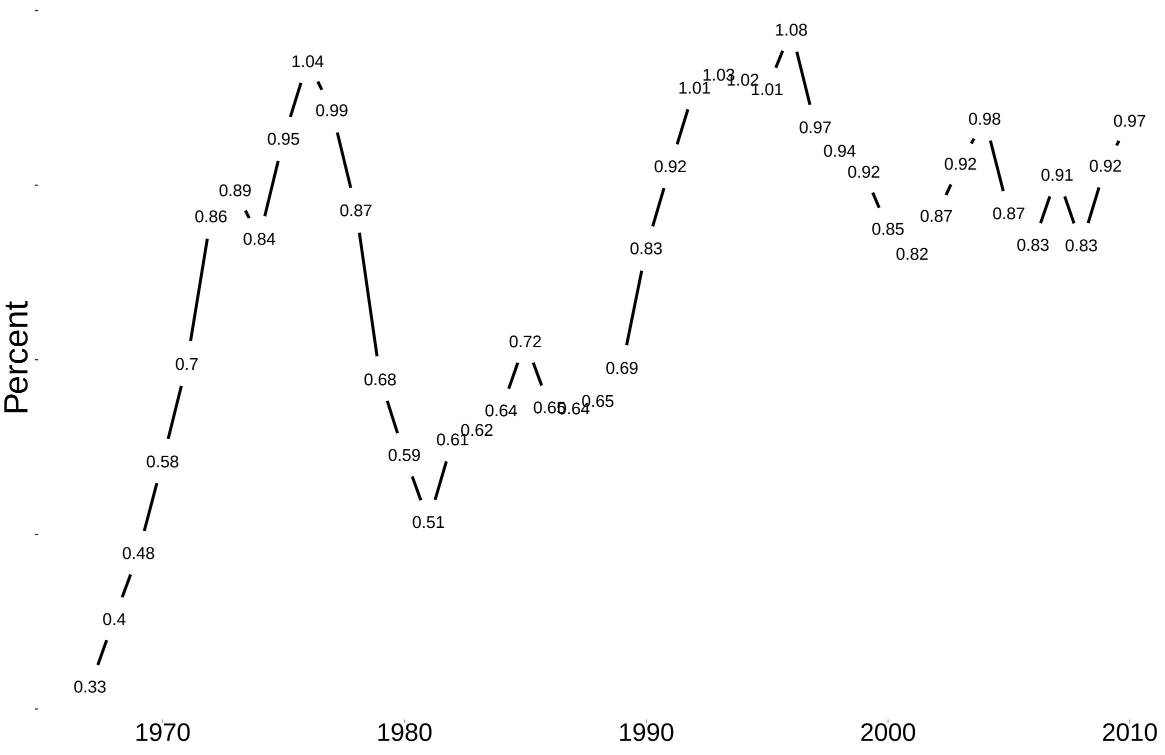

Fig. S4. Percent of sample who self-report intention to be a cop in each year of the TFS survey.

6. DV Measurement By Year

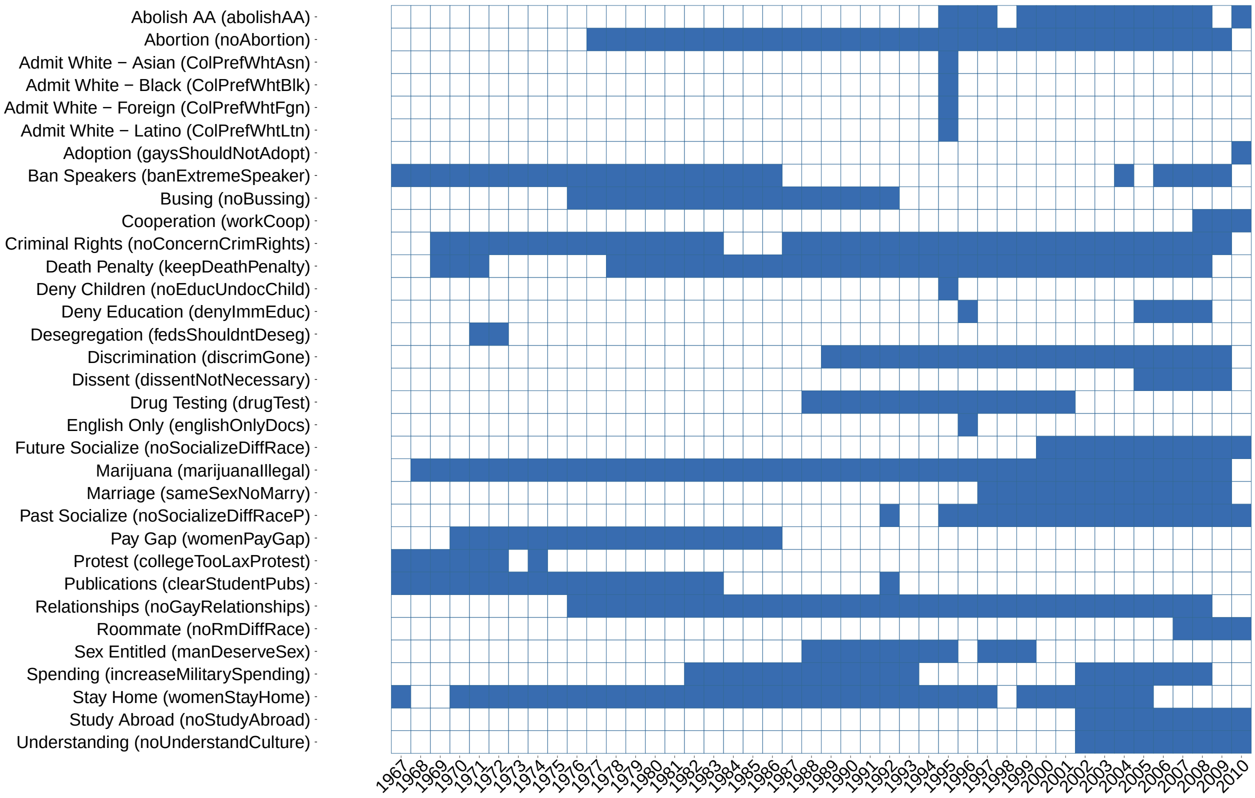

Fig. S5. Years DV Was Measured

7. Effect Heterogeneity by Sample Size

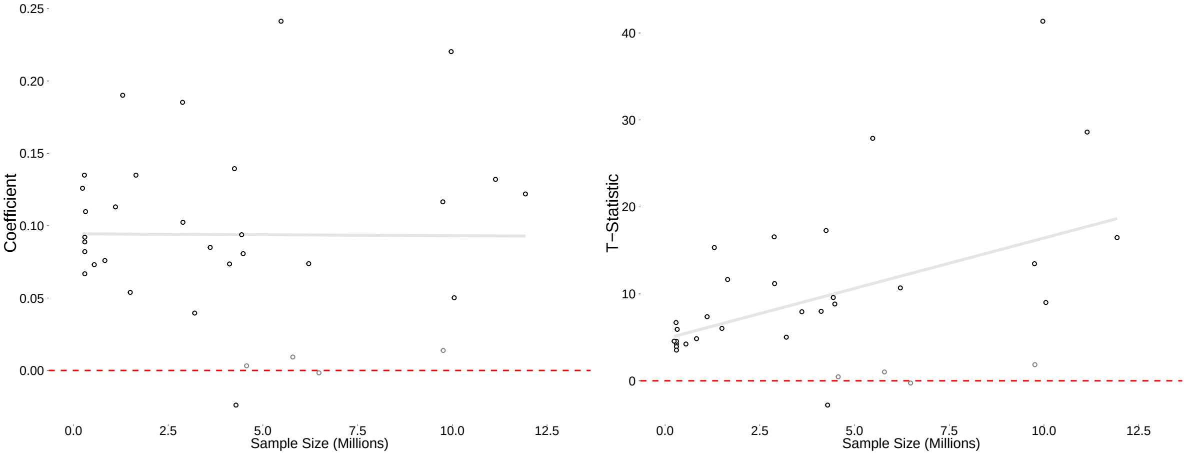

Fig. S6. Standardized Coefficients (Panel A) and T-statistics (Panel B) from fully specified models (i.e. from manuscript Figure 1, Panel A) plotted against sample size. Each point on the figure denotes a separate outcome test (of which there are 33). We find no clear pattern between the association and sample size / number of waves on which the items were asked nor between the presence / absence of statistical significance and sample size, though t-statistics do increase as sample size increases.

8. Alternate Modeling Specifications

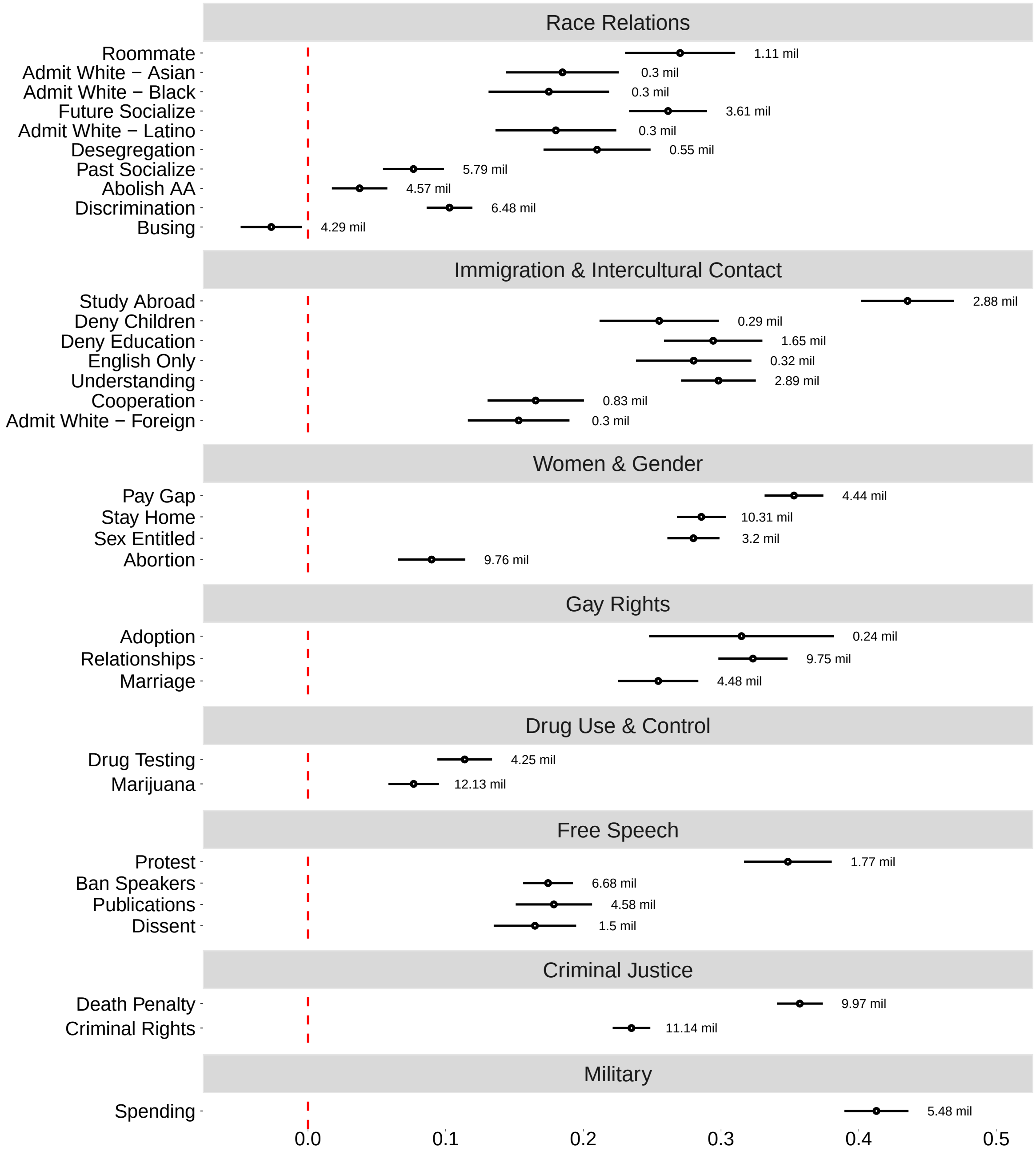

Fig. S7. Bivariate Model Results. Baseline comparison group = "all other." Coefficients presented are mean standardized. 95% CIs displayed.

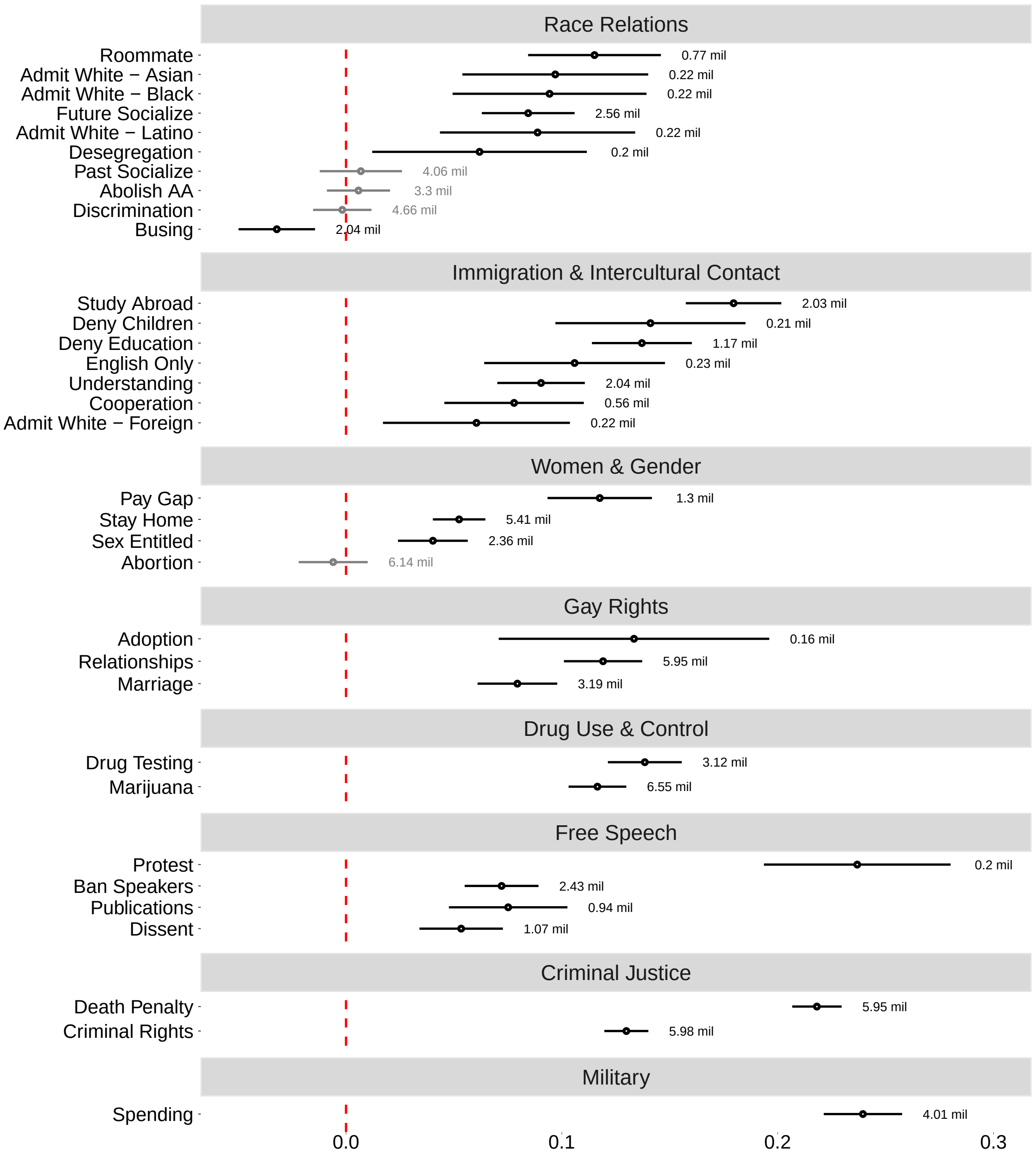

Fig. S8. List-wise Deletion for Missing Data Model Results. Baseline comparison group = “all other.” Mean standardized coefficients presented. 95% CIs displayed.

9. Survey and Questions

In Table S2 we display the mean, standard deviation, minimum values, and maximum values for each variable used in our analysis. Full question wordings for each DV can be found in Table 1 in the main manuscript.

Type	Variable	Min.	Max.	Mean	SD	Response Categories
DV	Discrimination	1.00	4.00	1.83	0.79	1=Disagree strongly to 4=Agree strongly
	Abolish AA	1.00	4.00	2.60	0.91	1=Disagree strongly to 4=Agree strongly
	Admit White - Black	-2.00	2.00	-0.05	0.35	-2=Pref Black over White to 2=Pref White over Black
	Admit White - Hispanics	-2.00	2.00	-0.04	0.35	-2=Pref Hispanic over White to 2=Pref White over Hispanic
	Admit White - Asians	-2.00	2.00	-0.03	0.31	-2=Pref Asian over White to 2=Pref White over Asian
	Busing	1.00	4.00	2.62	0.91	1=Disagree strongly to 4=Agree strongly
	Desegregation	1.00	4.00	2.46	0.90	1=Disagree strongly to 4=Agree strongly
	Past Socialize	1.00	3.00	1.35	0.54	1=Not at all to 3=Frequently
	Future Socialize	1.00	4.00	1.40	0.64	1=Not at all to 3=Frequently
	Roommate	1.00	4.00	2.05	0.92	1=No chance to 5=Really good chance
	Deny Education	1.00	4.00	2.47	1.01	1=Disagree strongly to 4=Agree strongly
	Deny Children	1.00	4.00	2.18	1.00	1=Disagree strongly to 4=Agree strongly
	English Only	1.00	4.00	2.41	1.04	1=Disagree strongly to 4=Agree strongly
	Study Abroad	1.00	4.00	2.19	1.01	1=No chance to 4=Pretty good chance
	Understanding	1.00	4.00	2.44	0.94	1=Not important to 4=Essential
	Cooperation	1.00	5.00	1.89	0.75	1=Lowest 10% to 5=Highest 10%
	Admit White - Foreign	-2.00	2.00	-0.31	0.66	-2=Pref FB over White to 2=Pref White over FB
	Stay Home	1.00	4.00	1.82	0.97	1=Disagree strongly to 4=Agree strongly
	Equal Pay	1.00	4.00	1.41	0.69	1=Disagree strongly to 4=Agree strongly
	Sex Entitled	1.00	4.00	1.44	0.81	1=Disagree strongly to 4=Agree strongly
	Abortion	1.00	4.00	2.37	1.16	1=Disagree strongly to 4=Agree strongly
	Prohibit Relations	1.00	4.00	2.10	1.06	1=Disagree strongly to 4=Agree strongly
	Marriage	1.00	4.00	2.30	1.12	1=Disagree strongly to 4=Agree strongly
	Adoption	1.00	4.00	1.85	1.01	1=Disagree strongly to 4=Agree strongly
	Marijuana	1.00	4.00	2.93	1.05	1=Disagree strongly to 4=Agree strongly
	Drug Testing	1.00	4.00	3.06	0.89	1=Disagree strongly to 4=Agree strongly
	Ban Speakers	1.00	4.00	1.96	0.92	1=Disagree strongly to 4=Agree strongly
	Publications	1.00	4.00	2.12	0.87	1=Disagree strongly to 4=Agree strongly
	Dissent	1.00	4.00	2.22	0.74	1=Disagree strongly to 4=Agree strongly
	Protest	1.00	4.00	2.45	0.85	1=Disagree strongly to 4=Agree strongly
	Spending	1.00	4.00	2.72	0.89	1=Disagree strongly to 4=Agree strongly
	Criminal Rights	1.00	4.00	2.87	1.03	1=Disagree strongly to 4=Agree strongly
	Death Penalty	1.00	4.00	2.06	0.86	1=Disagree strongly to 4=Agree strongly
IV	Police Career	0.00	1.00	0.01	0.09	1=Yes, 0=No
Controls	Ideology	1.00	5.00	3.10	0.79	1=Far right to 5=Far left
	Father Education	1.00	8.00	5.18	2.12	1=Grammar school or less to 8=Graduate school
	Mother Education	1.00	8.00	4.84	1.91	1=Grammar school or less to 8=Graduate school
	Income	1.00	3.00	1.87	0.80	1=Lowest tercile to 3=Highest tercile
	Female	0.00	1.00	0.53	0.50	1=Female, 0=Other
	First Gen	0.00	1.00	0.26	0.44	1=First-gen, 0=Other
	White	0.00	1.00	0.80	0.40	1=White, 0=Other
	Citizen	0.00	1.00	0.98	0.13	1=Citizen, 0=Other
	GPA	1.00	8.00	5.82	1.64	1=D to 8=A or A+
	Parent Cop	0.00	1.00	0.02	0.12	1=Yes, 0=No

Table S2. Descriptive statistics for all variables used across all analyses in full sample. For more on DV wordings, see Table 1 in the manuscript

Variable Name	Intending Officer	All Other	Other Civilian	Undecided
Discrimination	1.90 (0.83)	1.83 (0.79)	1.82 (0.78)	1.80 (0.76)
Abolish AA	2.63 (0.91)	2.60 (0.91)	2.60 (0.90)	2.58 (0.86)
Admit White - Black	0.01 (0.39)	-0.05 (0.35)	-0.05 (0.35)	-0.04 (0.33)
Admit White - Hispanic	0.02 (0.39)	-0.04 (0.34)	-0.04 (0.34)	-0.03 (0.33)
Admit White - Asian	0.03 (0.35)	-0.03 (0.31)	-0.03 (0.31)	-0.02 (0.30)
Busing	2.60 (0.93)	2.62 (0.91)	2.63 (0.90)	2.59 (0.87)
Desegregation	2.65 (0.88)	2.46 (0.90)	2.47 (0.90)	2.40 (0.88)
Past Socialize	1.39 (0.57)	1.35 (0.54)	1.35 (0.54)	1.35 (0.53)
Roommate	2.30 (0.99)	2.05 (0.92)	2.05 (0.92)	2.01 (0.88)
Deny Education	2.77 (1.05)	2.47 (1.01)	2.47 (1.01)	2.36 (0.97)
Deny Children	2.43 (1.05)	2.18 (0.99)	2.18 (0.99)	2.14 (0.99)
English Only	2.70 (1.07)	2.41 (1.04)	2.41 (1.03)	2.34 (1.00)
Study Abroad	2.63 (0.98)	2.19 (1.01)	2.19 (1.01)	2.07 (0.99)
Understanding	2.72 (0.92)	2.44 (0.93)	2.44 (0.93)	2.39 (0.92)
Cooperation	2.01 (0.79)	1.89 (0.75)	1.89 (0.75)	1.95 (0.74)
Admit White - Foreign	-0.22 (0.67)	-0.32 (0.66)	-0.32 (0.65)	-0.30 (0.65)
Stay Home	2.07 (0.99)	1.82 (0.97)	1.80 (0.96)	1.72 (0.92)
Equal Pay	1.65 (0.80)	1.41 (0.69)	1.40 (0.68)	1.35 (0.64)
Sex Entitled	1.65 (0.92)	1.44 (0.81)	1.43 (0.80)	1.38 (0.76)
Abortion	2.47 (1.12)	2.37 (1.16)	2.37 (1.16)	2.25 (1.14)
Prohibit Relations	2.41 (1.10)	2.10 (1.06)	2.08 (1.06)	1.93 (1.02)
Marriage	2.58 (1.11)	2.30 (1.12)	2.29 (1.12)	2.14 (1.09)
Adoption	2.17 (1.09)	1.85 (1.01)	1.84 (1.01)	1.72 (0.95)
Marijuana	2.99 (1.04)	2.93 (1.05)	2.93 (1.05)	2.80 (1.05)
Drug Testing	3.17 (0.89)	3.06 (0.89)	3.06 (0.89)	2.94 (0.90)
Ban Speakers	2.13 (0.97)	1.96 (0.92)	1.95 (0.92)	1.90 (0.89)
Publications	2.24 (0.87)	2.12 (0.87)	2.11 (0.86)	2.03 (0.84)
Dissent	2.34 (0.69)	2.22 (0.74)	2.22 (0.74)	2.17 (0.75)
Protests	2.72 (0.88)	2.45 (0.85)	2.45 (0.85)	2.31 (0.83)
Spending	2.42 (0.89)	2.06 (0.86)	2.04 (0.85)	1.96 (0.81)
Criminal Rights	2.92 (0.93)	2.71 (0.89)	2.71 (0.88)	2.61 (0.86)
Death Penalty	3.25 (0.94)	2.86 (1.03)	2.86 (1.03)	2.73 (1.03)

Table S3. Mean outcomes for all DVs across relevant comparison groups with standard deviations in parentheses.

10. Police Career Coefficient Comparison Ideology Coefficient

Outcome	Police Career Coef.	Ideology Min-Max (0/1) Coef.	Police Career / Ideology Ratio
Ban Speakers	0.07	0.99	0.07
Protest	0.19	1.66	0.11
Dissent	0.05	0.49	0.11
Publications	0.07	0.96	0.08
Admit White - Black	0.09	0.53	0.17
Admit White - Asian	0.09	0.46	0.20
Admit White - Latino	0.08	0.54	0.15
Desegregation	0.07	1.36	0.05
Future Socialize	0.08	0.37	0.23
Roommate	0.11	0.49	0.23
Study Abroad	0.19	0.50	0.37
Understanding	0.10	0.67	0.15
Deny Education	0.13	1.12	0.12
Deny Children	0.13	0.86	0.16
English Only	0.11	0.88	0.12
Cooperation	0.08	0.55	0.14
Admit White - Foreign	0.07	0.05	1.45
Sex Entitled	0.04	0.08	0.47
Stay Home	0.05	0.72	0.07
Pay Gap	0.09	0.53	0.18
Adoption	0.13	1.99	0.06
Marriage	0.08	2.07	0.04
Relationships	0.12	1.35	0.09
Marijuana	0.12	1.28	0.10
Drug Testing	0.14	0.89	0.16
Death Penalty	0.22	0.93	0.24
Criminal Rights	0.13	0.92	0.14
Spending	0.24	1.39	0.17
**Average**			**0.20**

Table S4. Ratio of Police Career Coefficients to Ideology Coefficients For all 28 Positive and Statistically Significant Police Career Coefficient Outcome Tests. Outcome is mean-standardized, Police Career is binary (0/1), and Ideology is rescaled between 0-1 (min-max) so to be on the same scale as Police Career. Output are from models where the comparison group for Police Career intention is all other respondents: "baseline = all other."

11. Comparison With Prior Studies Using TFS Data

Article	Journal	Variable	Table	Coef
Mendelberg, McCabe, and Thal (2017) (1)	*American Journal of Political Science*	*49-59% Affluent*	Table 1, Column 1	0.09
Mendelberg, McCabe, and Thal (2017) (1)	*American Journal of Political Science*	*More Than 59% Affluent*	Table 1, Column 1	0.17
Chan and Raychaudhuri (2025) (2)	*Journal of Politics*	*Enrolled in Ethnic Studies Course*	Table A1, Column 1	0.059
Mijs (2023) (3)	*Research in Social Stratification and Mobility*	*Different-race Roommate*	Table 3 Column 1	0.12
Herrera et al. (2013) (4)	*Review of Higher Education*	*Participated in a Study-Abroad Program*	Table 2, Column 1	0.062
**Our Study**		***Police Career Intention***	**Average of Figure 1, Panel A**	**0.11**

Table S5. Standardized coefficients for main effects in other published studies using TFS data in comparison to the average standardized coefficient across statistically significant and positive outcome tests for *Police Career* in our study.

12. Police Career Coefficient Comparison With Prior Research on Law Enforcement Officer Attitudes

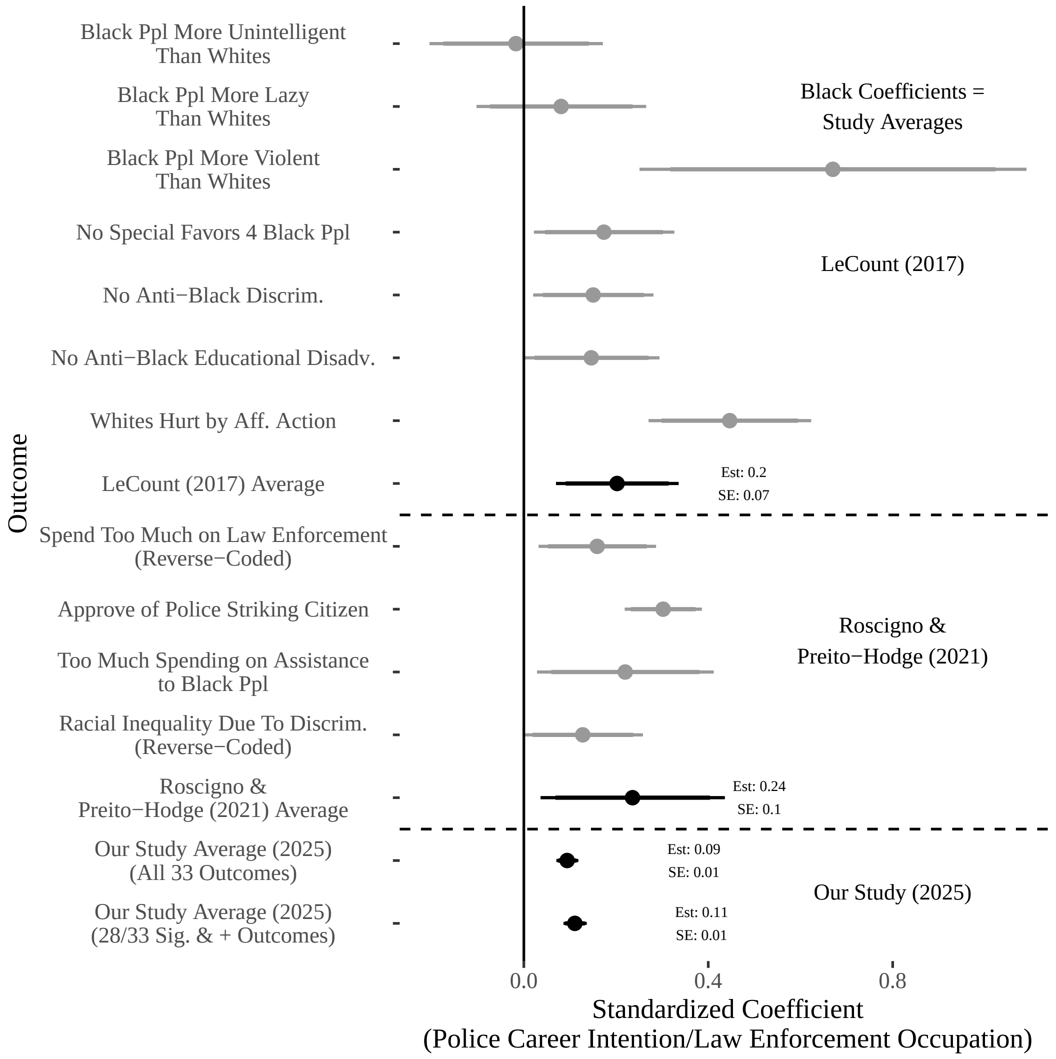

Fig. S9. Comparing average of study coefficient to coefficients from prior studies. The y-axis is the outcome of interest (or study average) analyzed across LeCount (2017) and Roscigno and Preito-Hodge (2021). The x-axis is the standardized coefficient for law enforcement occupation (for LeCount and R&P) or *Police Career* intention (for our study). Dashed horizontal lines separate the studies (from top to bottom: LeCount, Roscigno & Preito-Hodge, and our Study). Grey coefficients are standardized coefficients based on individual outcome tests, black coefficients are the average of standardized coefficients based on individual outcome tests within-study. Study averages are produced by averaging within-study standardized coefficients and standard errors. Annotations near coefficients denote the standardized coefficient size and standard error for the study averages. Our study coefficient averages are from models where the comparison is between those intending a police career and all other respondents ("baseline = all other"). We choose coefficient estimates where the reference category for *Police Career* is all other respondents for comparability's sake since LeCount (2017) and R&P (2021) compare those with a law enforcement occupation with all other members of the public. 95% CIs displayed from robust standard errors.

Figure S9 characterizes a comparison of standardized coefficients from LeCount (2017) and Roscigno and Preito-Hodge (2021) (5, 6) with standardized coefficients from our own study. LeCount (2017) and Roscigno and Preito-Hodge (2021) (hereafter R&P) both use nationally representative public opinion data of adults from the General Social Survey to evaluate the association between law enforcement occupation and right-leaning attitudes on race and criminal justice. In LeCount (2017), we replicate their fully-specified models on Table I, Table II, Table III, and the first outcome on Table IV ("whites hurt by affirmative action") in their published paper. In R&P (2021), we replicate their fully-specified models on Tables 1 and Tables 2 in their published paper. The average standardized coefficient size characterizing the relationship between being a law enforcement officer and right-leaning attitudes toward social groups in LeCount (2017) is 0.2. The average standardized coefficient size characterizing the relationship between being a law enforcement officer

and right-leaning attitudes toward social groups, police spending, and police use of force is 0.24. Thus, the average coefficient characterizing the relationship between being a law enforcement officer and right-leaning attitudes toward social groups and the police is 0.22 in LeCount (2017) and R&P (2021). The standardized *Police Career* coefficient is 0.09 for all 33 outcome tests and 0.11 for all 28 positive and statistically significant coefficients from the 33 outcome tests, equivalent to 41-50% of the average coefficient in prior studies comparing right-leaning attitudes between law enforcement officers and the general public.

We replicate LeCount (2017) and R&P (2021) to compare our coefficients characterizing the association between *Police Career* intention and right-leaning attitudes toward social groups with preexisting research on the relationship between law enforcement occupation and right-leaning attitudes toward social groups. We focus on these two studies specifically because a) they use publicly available General Social Survey data that allows us to effectively replicate their findings and models and b) are the most analogous to our study. To the best of our knowledge, there are four other studies we could have ostensibly incorporated in the comparison analysis on Figure S9 aside from LeCount (2017) and R&P (2021). However, we do not include them for several reasons: 1) Sidanius, Liu, Shaw, and Pratto (1994), which compares social dominance attitudes between police officers, public defenders, and jurors, but we do not include this study since it does not use publicly available data we can use to replicate the study's models and the study compares police officers to public defenders and jurors whereas our analysis compares those who intend to be a police officer with those who do not (that is, the rest of the late adolescent public entering college); 2) Sidanius, Pratto, Sinclair and van Laar (1996), which analyzes the relationship between self-reported attractiveness of working as a law enforcement officer and social dominance orientation among UCLA undergraduates, but we do not include this study since the study and the journal does not make the data publicly available so we can replicate their models; 3) Parker, Morin, and Rohal (2017), which is a Pew Research Center-led study analyzing differences in attitudes toward social groups between police and the general public, but we do not include this study since the Pew Research Survey does not make their survey of police publicly available; 4) Ba, Kaplan, Knox, Komisarchik, Lanzalotto, Mariman, Mummolo (2025), but we do not include this study since the outcome that may be of interest from this study (political partisan registration) is not analogous to our outcome(s) of interest, which typically characterize attitudes toward marginalized social groups.

Given LeCount (2017) and R&P (2021) do not provide replication code in their articles or housed on their respective journals, we attempt to replicate their findings and model specifications to the best of our ability since they use publicly available General Social Survey data, but there are differences in our replication we will note that are relevant to readers.

First, instead of using logistic regression models like LeCount (2017) and R&P (2021), we use linear models to assess the relationship between law enforcement occupation and right-leaning attitudes on race in order to facilitate interpretability and comparability with our estimates which rely on linear models.

Second, we standardize all outcomes across LeCount (2017) and R&P (2021) to facilitate comparability with our standardized coefficient estimates for our study. To do this, we subtract the mean of each outcome by each respondent-level outcome quantity and divide this quantity by the standard deviation of the outcome $(\frac{X_i - \mu(X_i)}{SD(X_i)})$

Third, R&P (2021) includes an interaction between law enforcement occupation and time (in years) in their models when the interaction is statistically significant, but not when the interaction is statistically insignificant. Since we are only concerned with the first-order association between law enforcement occupation and right-leaning attitudes in our study, we do not include this interaction in our replication of R&P (2021) for the sake of comparability with our study (but we still adjust for time in years).

Fourth, LeCount (2017) separates their analyses by race (white/Black). We pool these subsamples to facilitate comparability with our analysis. This does not change the substantive conclusions of LeCount (2017) since the majority of law enforcement officers in their sample is white and our re-analysis with the pooled sample approximates their findings concerning white law enforcement officers.

Fifth, we do not analyze three outcomes LeCount (2017) analyzes in our replication: (1) "resents special favors to African-Americans"; (2) "expects family to suffer racial discrimination"; (3) "claims

workplace racial discrimination." This is because outcome (1) requires the use of a different model specification due to missing data and the fact it is only available in one wave of the GSS; and outcomes (2)-(3) are not analogous outcomes to the ones we analyze in our main study since they are not about right-leaning attitudes toward other social groups or right-leaning attitudes about the status of other social groups, but rather personal perceptions/experiences of discrimination. Although outcome (1) is an analogous outcome to the outcomes we analyze in our study, excluding it should not change our conclusions with respect to our comparison between our study coefficients and the average of LeCount's study coefficients since law enforcement occupation is similarly associated with (1) as the "no special favors" outcome we do analyze on Figure S9 in LeCount (2017).

13. Weighting

We opt to not use the survey weights in our main regression analyses for a few reasons. First, there is an enormous amount of missingness on the weight variable in the pooled dataset. 4.3 million respondents are missing a weight (33% of the sample). This missingness varies year by year with anywhere from 3% to 100% of any given year's sample missing weights. Second, HERI has adjusted the weighting procedure several times over the years. They note that institutional stratification schemes were adjusted in 1969, 1971, 1975, 2001, and 2008, which leads to revisions of the weighting procedures over time and thus inconsistency in how the weights are adjusting the sample across our pooled dataset. Finally, some of the weights are very large. 38% of the sample has calculated weights of over 5 and 12% over 10 which can introduce strong design effects into our analyses. Nevertheless, we re-estimate our models using the survey weights in three different ways. First, we run our models using the raw weight variable with missingness (”Original”), which listwise deletes those observations without weights. Second, we re-estimate our models with the same weight variable that recodes missing weights as 1 (no adjustment) to those observations with missingness (”Imputed”). Finally, we re-estimate our models one final time this time using those same weights but also trimming them at 5 to avoid strong design effects in the adjustments (”Imputed + Trimmed”). We plot these estimates together with the original unweighted estimated below in Figure S10. For more on weighting methods see: (https://www.heri.ucla.edu/PDFs/CIRP_Reliability_Validity.pdf).

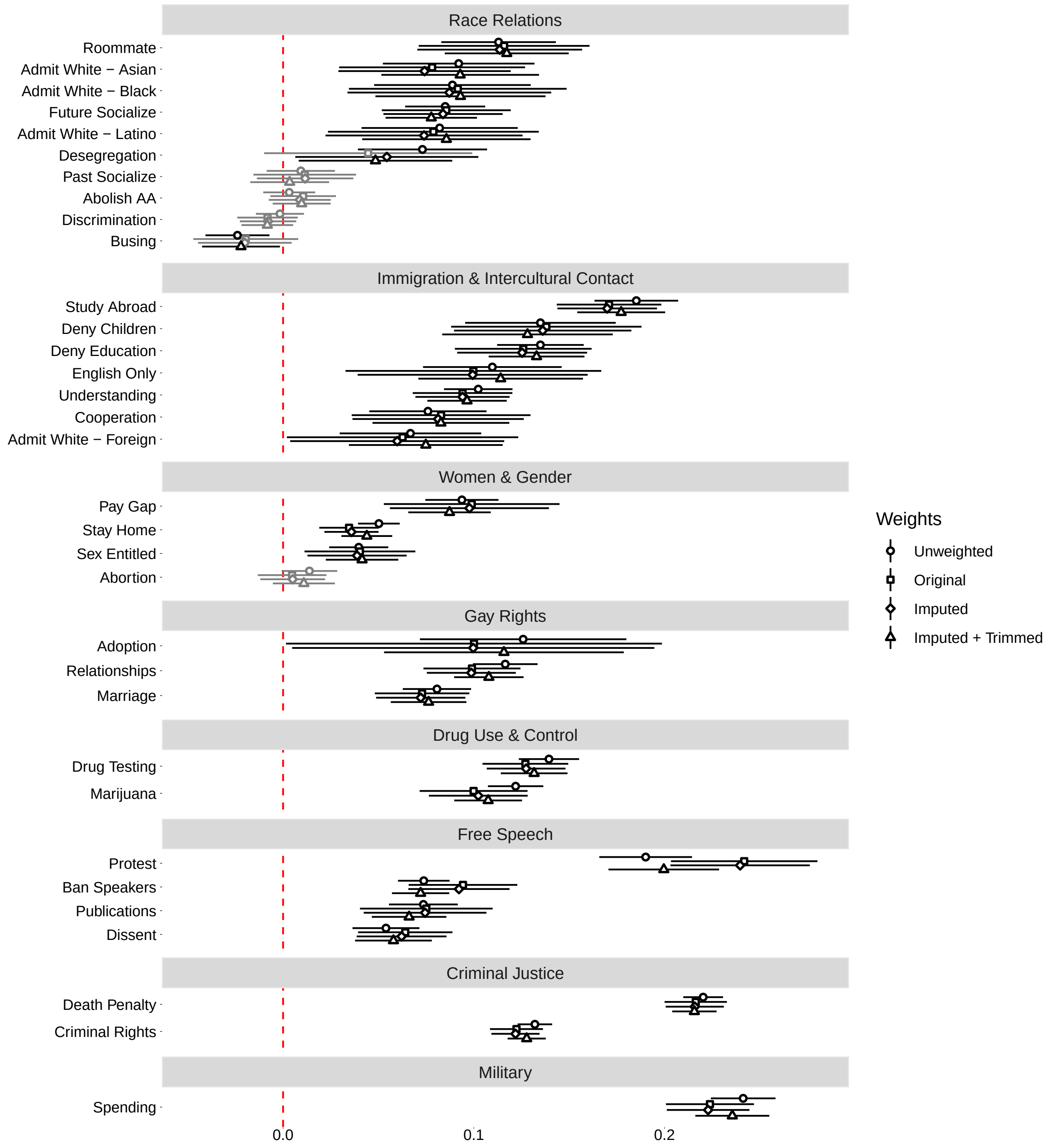

Fig. S10. Modeling results using different weighting schemes. Mean standardized coefficients presented. 95% CIs displayed.

14. Ordered Probit Models

Category	Variable	Coefficient	SE
Race Relations	Abolish AA	0.00	0.01
	Admit White - Black	0.17	0.03
	Admit White - Asian	0.18	0.03
	Admit White - Latino	0.17	0.03
	Discrimination	0.00	0.00
	Busing	-0.03	0.01
	Desegregation	0.08	0.02
	Future Socialize	0.07	0.01
	Past Socialize	0.00	0.01
	Roommate	0.11	0.01
Immigration	Study Abroad	0.19	0.01
	Understanding	0.12	0.01
	Deny Education	0.16	0.01
	Deny Children	0.15	0.02
	English Only	0.12	0.02
	Cooperation	0.08	0.01
	Admit White - Foreign	0.08	0.02
Women	Sex Entitled	-0.00	0.01
	Stay Home	0.03	0.00
	Pay Gap	0.07	0.01
	Abortion	0.03	0.00
Gay Rights	Adoption	0.14	0.02
	Marriage	0.09	0.01
	Relationships	0.06	0.00
Drug Use	Marijuana	0.11	0.00
	Drug Testing	0.17	0.01
Free Speech	Ban Speakers	0.15	0.00
	Protest	0.20	0.01
	Dissent	0.06	0.01
	Publications	0.07	0.01
Race Criminal Justice	Death Penalty	0.30	0.00
	Criminal Rights	0.16	0.00
Race Relations	Spending	0.31	0.01

Table S6. Coefficient and standard errors from ordered probit models estimated with *polr* function in R. Estimation issues necessitated the removal of two covariates which had high inflation factors (*first_gen* and *citizen*) and year fixed effects. All remaining covariates (except for *job_cop*) are mean-standardized.

15. Scaled Outcomes

We scale outcomes over time by taking the average of all items in each category (race relations, immigration & intercultural contact, women & gender, gay rights, drug use & control, free speech, and criminal justice). Below in Figure S11 we show how each scale correlates with a variety of individual level factors including student high school GPA, ideology, and family income. Importantly, key covariates like GPA, ideology, and income are correlated with each of the indexed outcome categories in a relatively similar manner over time, suggesting our outcomes are measuring a similar concept (different dimensions of anti-egalitarian beliefs) over time. We show that there is relatively high year-to-year stability in correlation coefficients. We re-run our base model specification swapping out individual attitudinal outcomes for these scaled outcomes. We display the results of these in Figure S12.

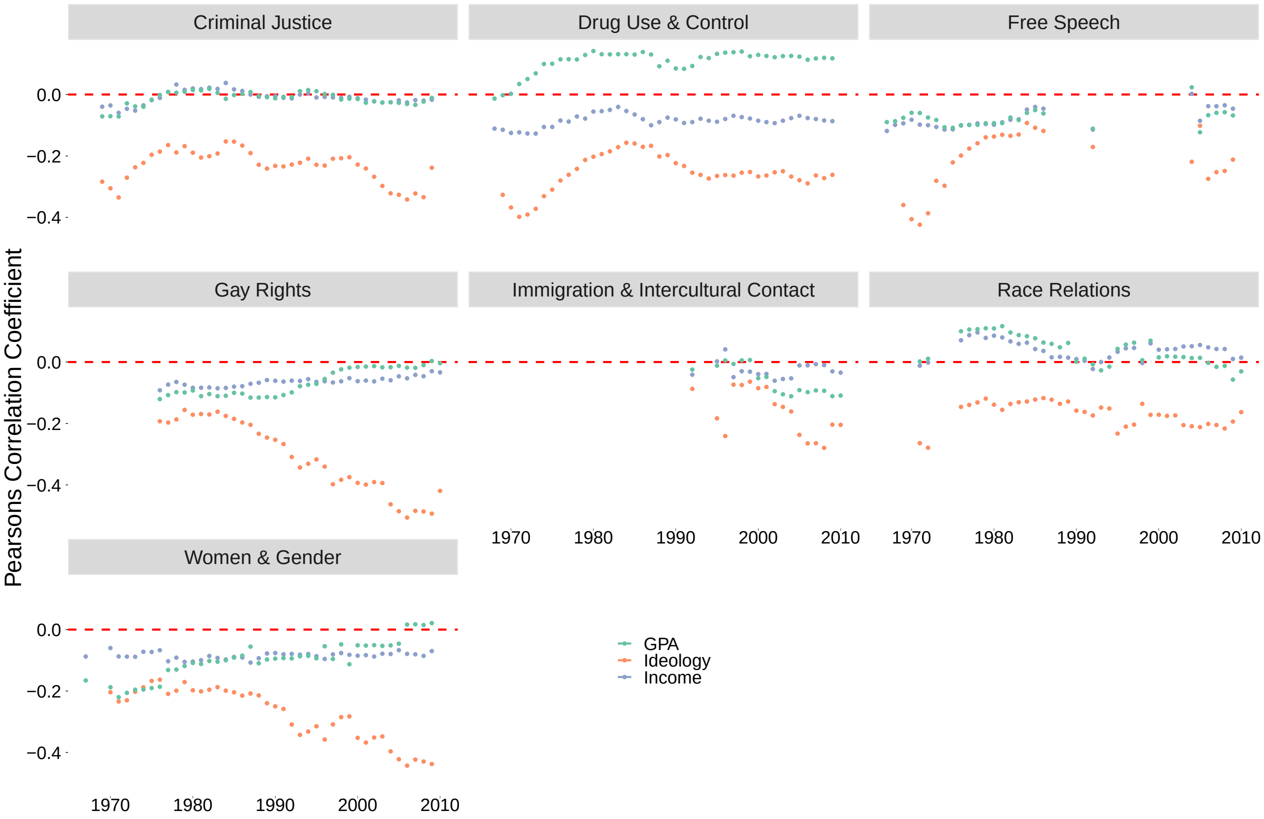

Fig. S11. Pearson's Correlation Coefficient for scaled outcomes and GPA, ideology, and family income over time.

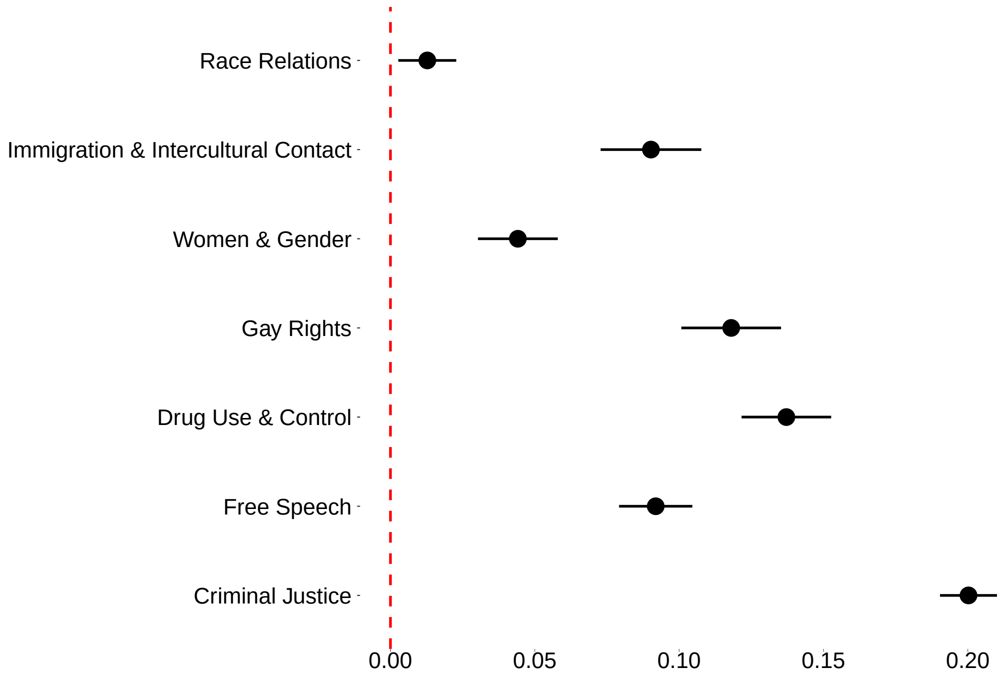

Fig. S12. Coefficient for police job intention IV using fully specified models and scaled outcomes with 95% confidence intervals. Outcomes are mean-standardized.

16. Additional Covariate Adjustment and Model Specifications

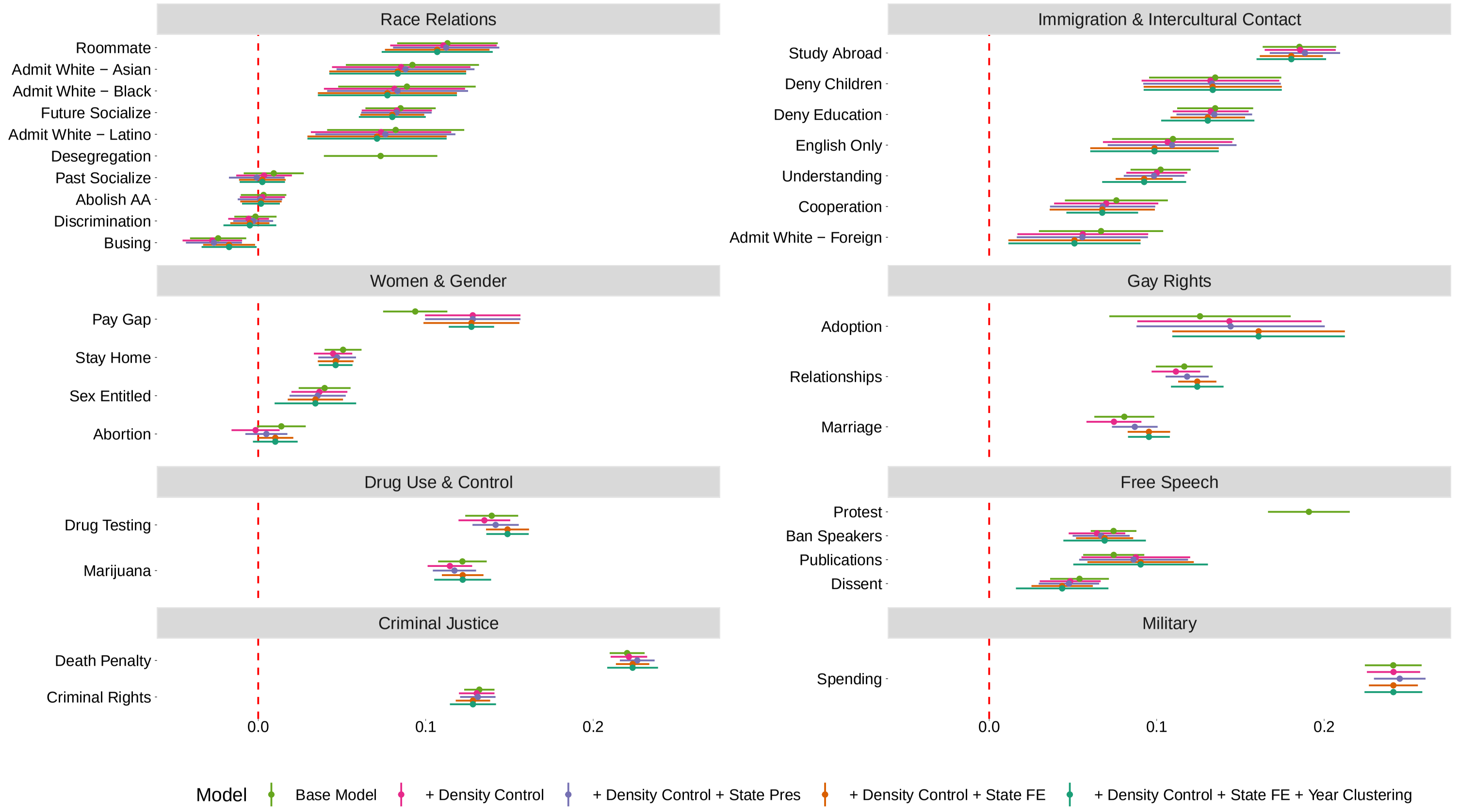

Fig. S13. Coefficients by model specification. We opt to not use geographic variables (e.g. density, state presidential election results, state fixed effects) in our main model because geographic identifiers were only included in TFS surveys starting in 1982 and thus these models drop 15 years of data and do not allow us to estimate some models as indicated by missing estimates in this figure. Outcomes are mean-standardized. Presidential election data from (7).

17. Binary Outcome

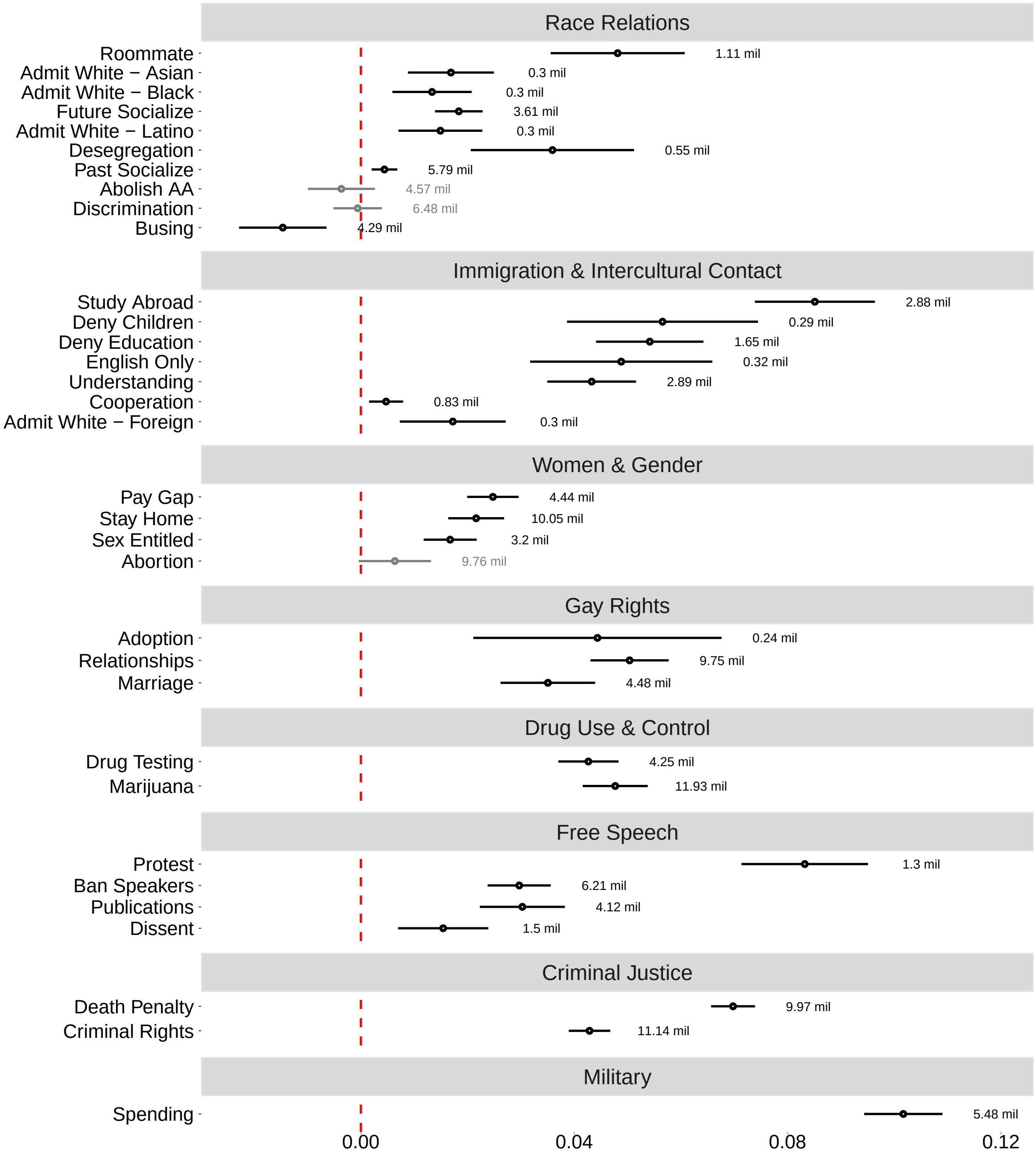

Fig. S14. Binary outcome analysis where the outcome is rescaled to a binary indicator where "1" is equal to whether or not the respondent reports any level of "agree" in the conservative direction of the outcome, "0" otherwise (baseline comparison = all other respondents). Annotation denotes sample size.

18. Extreme Category Binary Outcome

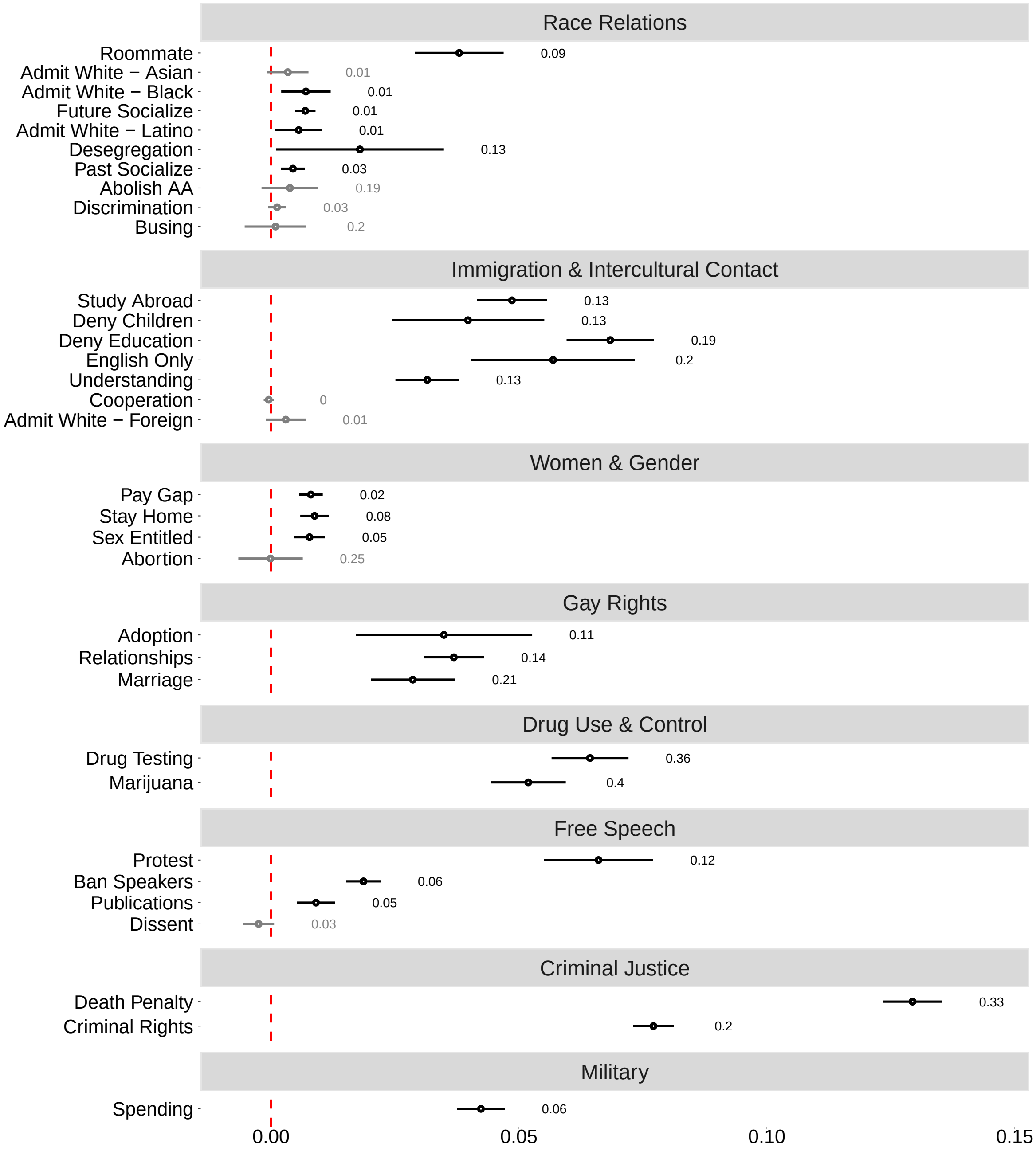

Fig. S15. Extreme outcomes analysis where the outcome is rescaled to a binary indicator where "1" is equal to the maximum value and "0" otherwise (baseline comparison = all other respondents). Annotation denotes outcome mean for those who do not intend a career in law enforcement.

19. Results By Year

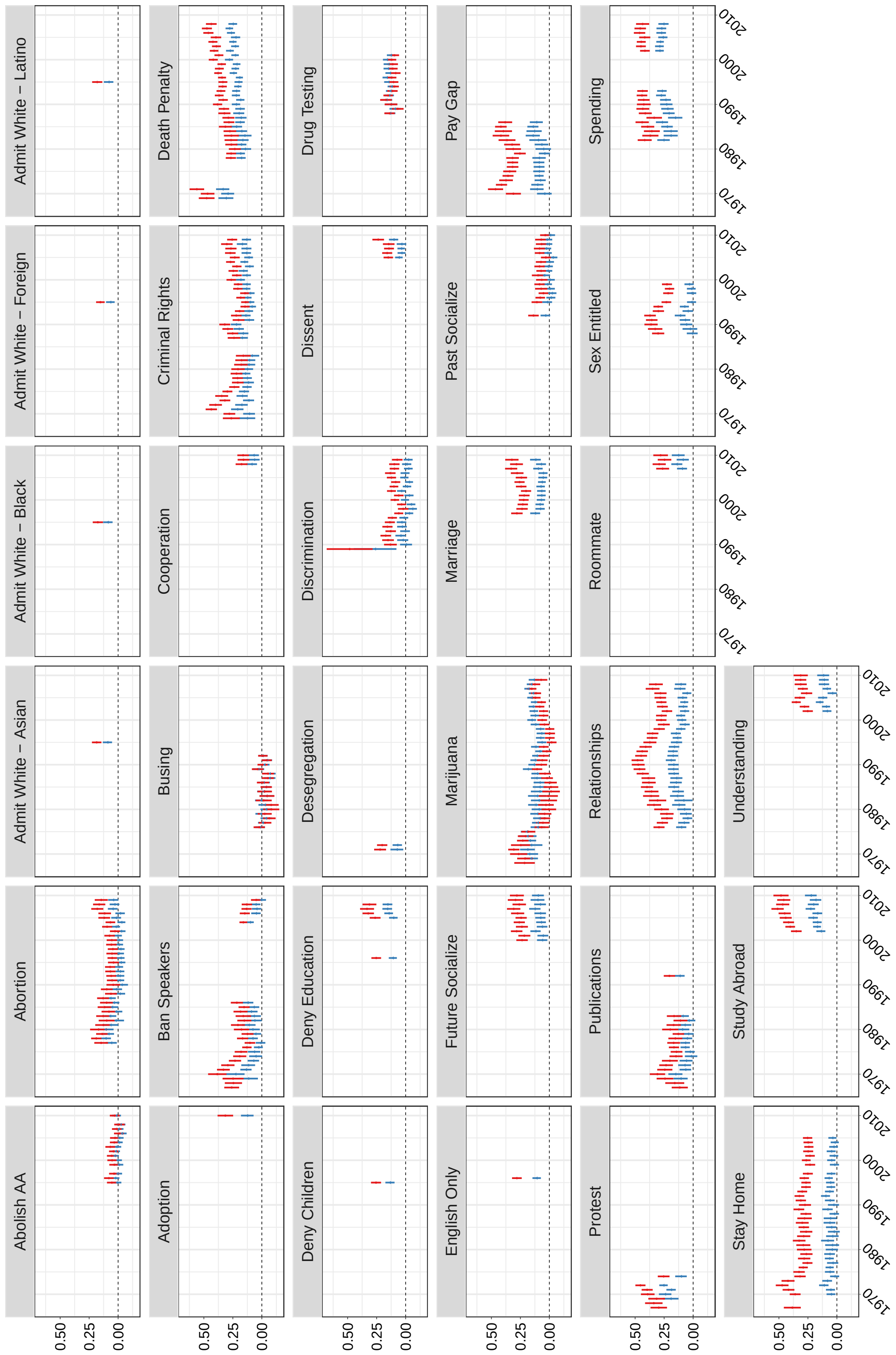

Fig. S16. Model Results by Year. Bivariate estimates in red and multivariate estimates in blue.

20. Survey Codebook

The Freshman Survey Codebook for "Late-adolescents entering college intending a career as police officers hold more right-leaning views than their peers."

This is a truncated codebook created for the purposes of displaying the order and full text of survey questions used in the main analyses for "Late-adolescents entering college intending a career as police officers hold more right-leaning views than their peers." By Tyler Reny, Marcel Roman, Benjamin Newman, and David Sears. This is only a subset of questions from the broader TFS Codebook, which we link to for reader's reference on the Higher Education Research Institute (HERI) public website (https://ucla.app.box.com/v/TFS-Trends-Survey-Items-xls).

This codebook maintains the order of the survey questions as specified in the TFS Codebookfile, so variables are not necessarily clustered by type (e.g. control variables, independent variables, dependent variables), but are displayed in the order that the TFS Codebook file displays the survey questions. Since this file only displays a subset of TFS questions (that is, the ones we use for our analyses), we refer readers to the TFS Codebook file on the public HERI website in order to get a fuller sense of all questions in the TFS (https://ucla.app.box.com/v/TFS-Trends-Survey-Items-xls).

Given the TFS is broken up in several files, we specify the FILE and then the VARIABLE name in square brackets []. Here is a list of the TFS files we use (in survey order as specified by the TFS codebook)

- DEMO: demographics (from the DEMOGRAPHICS POLICE.SAV file in the TFS)
- HS: high school characteristics (from the HIGH SCHOOL POLICE.SAV file in the TFS)
- PLANS: plans in college (from the PLANS POLICE.SAV file in the TFS)
- VIEWS: views on politics (from the VIEW POLICE.SAV file in the TFS)
- DISAGG: disaggregated file (from the DISAGG POLICE.SAV file in the TFS)

We also specify the use of each variable with reference to our main analyses in the main text of the paper or our supplementary analyses for further clarity in parentheses ().

[**DEMOS, YEAR**] Year survey was taken, this is meta-data and not an actual question. From 1966 to 2010. (We use this to assess effects over time in addition to adjust for temporal shocks.)

[**DEMOS, ACERECODE**] Code referring to the college/university/school the respondent is taking the survey from. This is meta-data and not an actual question. These are numeric codes, and there are 1839 unique codes across the entirety of the raw data. (We use this variable to adjust for school fixed effects.)

[**DEMOS, SELECTIVITY**] Code referring to the selectivity of the college/university/school the respondent is taking the survey from. This is meta-data and not an actual question.

[**DEMOS, INSTTYPE**] Code referring to the type of institution/college/university/school the respondent is taking the survey from. This is meta-data and not an actual question. (We use this variable to adjust for institution type and assess heterogeneity by institution type in the supplementary analyses)

- 1) University
- 2) 4-year
- 3) 2-year

[**DEMOS, HOMEZIP**] Self-reported zipcode of the student. Asked in TFS surveys after 1982. We use these codes to adjust for various geographic covariates in the supplementary analyses.

[**DEMOS, SEX**] What is your sex? (We use this variable to control for gender and assess heterogeneity of police career intention by gender)

- 1) Male
- 2) Female

[DEMOS, AGE2] How old will you be on December 31 of this year? (Respondent can report their age, we use this variable to control for age)

[DEMOS, RACEGROUP] Self-reported race/ethnicity of the student. (We use this variable to control for race and assess heterogeneity of police career intention by race)

- 1) American Indian
- 2) Asian
- 3) Black
- 4) Hispanic
- 5) White
- 6) Other
- 7) Two or more race/ethnicity

[DEMOS, CITIZEN] Self-reported citizenship status of the student. (We use this variable to control for immigration/citizenship status)

- 1) U.S. citizen
- 2) Permanent resident
- 3) International student
- 4) Neither/none of the above

[DEMOS, FATHEDUC] What is the highest level of formal education obtained by your parents/guardians? Parent/Guardian 1/Father. (We use this variable to control for parental education)

- 1) Grammar school or less
- 2) Some high school
- 3) High school graduate
- 4) Postsecondary school other than college
- 5) Some college
- 6) College degree
- 7) Some graduate school
- 8) Graduate degree

[DEMOS, MOTHEDUC] What is the highest level of formal education obtained by your parents/guardians? Parent/Guardian 1/Mother. (We use this variable to control for parental education)

- 1) Grammar school or less
- 2) Some high school
- 3) High school graduate
- 4) Postsecondary school other than college
- 5) Some college
- 6) College degree
- 7) Some graduate school
- 8) Graduate degree

[DEMOS, FIRSTGEN] First generation status coded based on the above questions (FATHEDUC and MOTHEDUC). Student is coded as "first-generation" if their parent(s) both have less than "some college." (We use this variable to control for first-generation status among the students that take the TFS survey)

- 1) No
- 2) Yes

[DEMOS, INCOME] What is your best estimate of your parents'/guardians' total income last year? Consider income from all sources before taxes. (We use this variable to control for income in our analyses)

- 1) Less than $6,000
- 2) $6000-$9999
- 3) Less than $10,000
- 4) $10,000-$14,999
- 5) Less than $15,000
- 6) $15,000-$19,999
- 7) $15,000-$24,999
- 8) $20,000-$24,999
- 9) $25,000-$29,999
- 10) $30,000 or more
- 11) $30,000-$34,999
- 12) $30,000-$39,999
- 13) $30,000-$59,999
- 14) $35,000-$39,999
- 15) $40,000 or more
- 16) $40,000-$49,999
- 17) $50,000 or more
- 18) $50,000-$59,999
- 19) $50,000-$99,999
- 20) $60,000-$74,999
- 21) $75,000-$99,999'
- 22) $100,000 or more
- 23) $100,000-$124,999
- 24) $100,000-$149,999
- 25) $125,000-$149,999
- 26) $150,000 or more
- 27) $150,000-$199,999
- 28) $200,000 or more
- 29) $200,000-$249,999
- 30) $250,000 or more
- 31) $250,000-$499,999
- 32) $500,000 or more
- 33) unknown

[**HS, HSGPA**] What was your average grade in high school? (We use this survey item to control for high school GPA in our models)

- 1) D
- 2) C
- 3) C+
- 4) B-
- 5) B
- 6) B+
- 7) A-
- 8) A or A+

[**HS, ACT25**] In the past year, how often have you socialized with someone of another racial/ethnic group? (We use this item to measure the extent to which respondents have socialized with someone of another racial/ethnic group as a "Race Relations" outcome)

- 1) Not at all
- 2) Occasionally
- 3) Frequently

[**HS, DIVRATE5_T**] How would you rate yourself in the following areas? Ability to work cooperatively with diverse peoples. (We use this item to measure the extent to which respondents are able to work cooperatively with diverse peoples as a "Immigration and Intercultural Relations" outcome)

- 1) Lowest 10
- 2) Below average
- 3) Average
- 4) Above average
- 5) Highest 10

[**PLANS, FUTACT16**] What is your best guess as to the chances that you will: Have a roommate of different race/ethnicity (We use this item to measure the extent to which respondents are to have a roommate of a different race/ethnicity as a "Race Relations" outcome)

- 1) No chance
- 2) Very little chance
- 3) Some chance
- 4) Very good chance

[**PLANS, FUTACT21**] What is your best guess as to the chances that you will: Participate in a study abroad program (We use this item to measure the extent to which respondents are to do a Study Abroad program as a "Immigration and Intercultural Relations" outcome)

- 1) No chance
- 2) Very little chance
- 3) Some chance
- 4) Very good chance

[**PLANS, FUTACT30**] What is your best guess as to the chances that you will: Socialize with someone of another racial/ethnic group (We use this item to measure the extent to which respondents are to socialize with someone of another racial/ethnic group as a "Race Relations" outcome)

- 1) No chance
- 2) Very little chance
- 3) Some chance
- 4) Very good chance

[**PLANS, GOAL14**] Please indicate the importance to you personally of each of the following: Improving my understanding of other countries and cultures

- 1) Not important
- 2) Somewhat important
- 3) Very important
- 4) Essential

[**VIEWS, POLIVIEW**] How would you characterize your political views? (We use this survey item to control for political ideology in our models)

- 1) Far right
- 2) Conservative
- 3) Middle of the road

- 4) Liberal
- 5) Far left

[**VIEWS, VIEW02**] Abortion should be abolished. (We use this item to measure disapproval of abortion as a “Women and Gender” outcome)

- 1) Disagree strongly
- 2) Disagree somewhat
- 3) Agree somewhat
- 4) Agree strongly

[**VIEWS, VIEW04**] Affirmative action in college admissions should be abolished. (We use this item to measure disapproval of affirmative action as a “Race Relations” outcome)

- 1) Disagree strongly
- 2) Disagree somewhat
- 3) Agree somewhat
- 4) Agree strongly

[**VIEWS, VIEW05**] Colleges have the right to ban extreme speakers from campus. (We use this item to measure approval of banning extreme speakers as a “Free Speech” outcome)

- 1) Disagree strongly
- 2) Disagree somewhat
- 3) Agree somewhat
- 4) Agree strongly

[**VIEWS, VIEW07**] Dissent is a critical component of the political process. (We use this item to measure disapproval of dissent as a “Free Speech” outcome)

- 1) Disagree strongly
- 2) Disagree somewhat
- 3) Agree somewhat
- 4) Agree strongly

[**VIEWS, VIEW08**] Employers should be allowed to require drug testing of employees or job applicants (We use this item to measure approval of drug testing as a “Drug Use and Control” outcome)

- 1) Disagree strongly
- 2) Disagree somewhat
- 3) Agree somewhat
- 4) Agree strongly

[**VIEWS, VIEW09**] Federal military spending should be increased (We use this item to measure approval of military spending as a “Military” outcome)

- 1) Disagree strongly
- 2) Disagree somewhat
- 3) Agree somewhat
- 4) Agree strongly

[**VIEWS, VIEW10**] Gays and lesbians should have the legal right to adopt a child. (We use this item to measure disapproval of gay/lesbian adoption as a “Gay Rights” outcome)

- 1) Disagree strongly

- 2) Disagree somewhat
- 3) Agree somewhat
- 4) Agree strongly

[VIEWS, VIEW13] It is important to have laws prohibiting homosexual relationships. (We use this item to measure approval of laws prohibiting homosexual relationships as a "Gay Rights" outcome)

- 1) Disagree strongly
- 2) Disagree somewhat
- 3) Agree somewhat
- 4) Agree strongly

[VIEWS, VIEW14] Just because a man thinks that a woman has 'led him on' does not entitle him to have sex with her (We use this item to measure approval of sex entitlement as a "Women and Gender" outcome)

- 1) Disagree strongly
- 2) Disagree somewhat
- 3) Agree somewhat
- 4) Agree strongly

[VIEWS, VIEW15] Marijuana should be legalized (We use this item to measure disapproval of marijuana legalization as a "Drug Use and Control" outcome)

- 1) Disagree strongly
- 2) Disagree somewhat
- 3) Agree somewhat
- 4) Agree strongly

[VIEWS, VIEW18] Racial discrimination is no longer a major problem in America (We use this item to measure disapproval of racial discrimination being a problem as a "Race Relations" outcome)

- 1) Disagree strongly
- 2) Disagree somewhat
- 3) Agree somewhat
- 4) Agree strongly

[VIEWS, VIEW20] Same-sex couples should have the right to legal marital status (We use this item to measure disapproval of same-sex marriage as a "Gay Rights" outcome)

- 1) Disagree strongly
- 2) Disagree somewhat
- 3) Agree somewhat
- 4) Agree strongly

[VIEWS, VIEW21] The activities of married women are best confined to the home and family. (We use this item to measure approval of the notion that women are best confined to the home as a "Women and Gender" outcome)

- 1) Disagree strongly
- 2) Disagree somewhat
- 3) Agree somewhat
- 4) Agree strongly

[VIEWS, VIEW23] The death penalty should be abolished (We use this item to measure disapproval of death penalty abolishment as a "Criminal Justice" outcome)

- 1) Disagree strongly

- 2) Disagree somewhat
- 3) Agree somewhat
- 4) Agree strongly

[**VIEWS, VIEW28**] There is too much concern in the courts for the rights of criminals (We use this item to measure approval of the notion that there is too much concern in the courts for the rights of criminals as a “Criminal Justice” outcome)

- 1) Disagree strongly
- 2) Disagree somewhat
- 3) Agree somewhat
- 4) Agree strongly

[**VIEWS, VIEW30**] Undocumented immigrants should be denied access to public education (We use this item to measure approval of the notion that undocumented immigrants should be denied access to public education as a “Immigration and Intercultural Relations” outcome).

- 1) Disagree strongly
- 2) Disagree somewhat
- 3) Agree somewhat
- 4) Agree strongly

[**VIEWS, VIEW39**] Women should receive the same salary and opportunities for advancement as men in comparable positions. (We use this item to measure disapproval of the notion that women should receive the same salary and opportunities for advancement as men as a “Women and Gender” outcome).

- 1) Disagree strongly
- 2) Disagree somewhat
- 3) Agree somewhat
- 4) Agree strongly

[**VIEWS, VIEWS04_T**] Busing is O.K. if it helps to achieve racial balance in the schools. (We use this item to measure disapproval of the notion that busing is good as a “Race Relations” outcome).

- 1) Disagree strongly
- 2) Disagree somewhat
- 3) Agree somewhat
- 4) Agree strongly

[**VIEWS, VIEWS15_T**] Most college officials have been too lax in dealing with students protest on campus.” (We use this item to measure approval of the notion that colleges have been lax in dealing with student protest as a “Free Speech” outcome)

- 1) Disagree strongly
- 2) Disagree somewhat
- 3) Agree somewhat
- 4) Agree strongly

[**VIEWS, VIEWS21_T**] Student publications should be cleared by college officials (We use this item to measure approval of the notion student publications should be cleared by college officials as a “Free Speech” outcome)

- 1) Disagree strongly
- 2) Disagree somewhat
- 3) Agree somewhat
- 4) Agree strongly

[VIEWS, VIEWS27_T] The federal government is not doing enough to promote school desegregation (We use this item to measure approval of the Federal government is not doing enough to promote desegregation as a “race relations” outcome)

- 1) Disagree strongly
- 2) Disagree somewhat
- 3) Agree somewhat
- 4) Agree strongly

[VIEWS, VIEW9502_N] Children of undocumented immigrants should be denied access to public education (We use this item to measure approval of denying access to public education for undocumented immigrants as a “Immigration and Intercultural Relations” outcome)

- 1) Disagree strongly
- 2) Disagree somewhat
- 3) Agree somewhat
- 4) Agree strongly

[VIEWS, VIEW96_N] All official federal and state documents should be printed in English only (we use this item to measure approval of English only policy as a “immigration and intercultural relations” outcome.)

- 1) Disagree strongly
- 2) Disagree somewhat
- 3) Agree somewhat
- 4) Agree strongly

[VIEWS, ADMITCOL9504] How much consideration should college admission officers give to Foreign Students? (we use this item to measure preferences for admitting whites vs other groups as “Race Relations” or “Immigration and Intercultural Relations” outcome.)

- 1) None
- 2) Some
- 3) A lot

[VIEWS, ADMITCOL9506] How much consideration should college admission officers give to African-Americans? (we use this item to measure preferences for admitting whites vs other groups as “Race Relations” or “Immigration and Intercultural Relations” outcome.)

- 1) None
- 2) Some
- 3) A lot

[VIEWS, ADMITCOL9508] How much consideration should college admission officers give to Asian-Americans? (we use this item to measure preferences for admitting whites vs other groups as “Race Relations” or “Immigration and Intercultural Relations” outcome.)

- 1) None
- 2) Some
- 3) A lot

[VIEWS, ADMITCOL9509] How much consideration should college admission officers give to Hispanics/Latinos? (we use this item to measure preferences for admitting whites vs other groups as “Race Relations” or “Immigration and Intercultural Relations” outcome.)

- 1) None
- 2) Some
- 3) A lot

[**VIEWS, ADMITCOL9510**] How much consideration should college admission officers give to Whites/Caucasians? (we use this item to measure preferences for admitting whites vs other groups as "Race Relations" or "Immigration and Intercultural Relations" outcome.) 1) None; 2) Some; 3) A Lot

[**DISAGG, SCAREER**] Please indicate your intended career (we use this item to measure the intended career of respondents in our main analyses). Equal to 1 if the respondent indicates they are a "law enforcement officer," 0 otherwise.

[**DISAGG, FCAREER**] Please indicate your father's career (we use this item to measure the career of parents of respondents in our main analyses). Equal to 1 if the respondent indicates their father OR mother is a "law enforcement officer," 0 otherwise.

[**DISAGG, MCAREER**] Please indicate your mother's career (we use this item to measure the career of parents of respondents in our main analyses). Equal to 1 if the respondent indicates their father OR mother is a "law enforcement officer," 0 otherwise.

References

1. T Mendelberg, KT McCabe, A Thal, College socialization and the economic views of affluent americans. *Am. J. Polit. Sci.* **61**, 606–623 (2017).
2. NKM Chan, T Raychaudhuri, Race, diversity, and the development of political attitudes on college campuses. *J. Polit.* (forthcoming).
3. JJB Mijs, Learning about inequality in unequal america: How heterogeneity in college shapes students' beliefs about meritocracy and racial discrimination. *Res. Soc. Stratif. Mobil.* **85** (2023).
4. FA Herrera, JC Garibay, GA Garcia, MP Johnston, Documenting attitudes toward undocumented immigrant access to public education: A multilevel analysis. *The Rev. High. Educ.* **36**, 513–549 (2013).
5. RJ LeCount, More black than blue? comparing the racial attitudes of police to citizens. *Sociol. Forum* **32**, 1051–1072 (2017).
6. VJ Roscigno, K Preito-Hodge, Racist cops, vested "blue" interests, or both? evidence from four decades of the general social survey. *Socius* **7**, 1–13 (2021).
7. S Amlani, C Algara, Partisanship nationalization in american elections: Evidence from presidential, senatorial, gubernatorial elections in the u.s. counties, 1872–2020. *Elect. Stud.* **73** (2021).
